# ISG15 accelerates acute kidney injury and the subsequent AKI-to-CKD transition by promoting TGFβR1 ISGylation

**DOI:** 10.7150/thno.95796

**Published:** 2024-07-22

**Authors:** Na Cui, Chengyu Liu, Xiang Tang, Liangliang Song, Zixuan Xiao, Chen Wang, Yancai Wu, Yihao Zhou, Chentai Peng, Yuxia Liu, Ling Zheng, Xinran Liu, Kun Huang, Hong Chen

**Affiliations:** 1 Tongji School of Pharmacy, Huazhong University of Science and Technology, Wuhan, China, 430030.; 2 Department of Transfusion Medicine, Wuhan Hospital of Traditional Chinese and Western Medicine, Tongji Medical College, Huazhong University of Science and Technology, Wuhan, China, 430000.; 3 ISA Wenhua Wuhan High School, Fenglin Road, Junshan New Town, Wuhan Economics & Technological Development Zone, Wuhan, Hubei, China, 430119.; 4 Hubei Key Laboratory of Cell Homeostasis, College of Life Sciences, Wuhan University, Wuhan, China, 430072.

**Keywords:** ISG15, TGFβR1, acute kidney injury, fibrosis, AKI-to-CKD transition

## Abstract

**Rationale:** Acute kidney injury (AKI) has substantial rates of mortality and morbidity, coupled with an absence of efficacious treatment options. AKI commonly transits into chronic kidney disease (CKD) and ultimately culminates in end-stage renal failure. The interferon-stimulated gene 15 (ISG15) level was upregulated in the kidneys of mice injured by ischemia-reperfusion injury (IRI), cisplatin, or unilateral ureteral obstruction (UUO), however, its role in AKI development and subsequent AKI-to-CKD transition remains unknown.

**Methods:** *Isg15* knockout (*Isg15* KO) mice challenged with bilateral or unilateral IRI, cisplatin, or UUO were used to investigate its role in AKI. We established cellular models with overexpression or knockout of ISG15 and subjected them to hypoxia-reoxygenation, cisplatin, or transforming growth factor- β1 (TGF-β1) stimulation. Renal RNA-seq data obtained from AKI models sourced from public databases and our studies, were utilized to examine the expression profiles of ISG15 and its associated genes. Additionally, published single cell RNA-seq data from human kidney allograft biopsies and mouse IRI model were analyzed to investigate the expression patterns of ISG15 and the type I TGF-β receptor (TGFβR1). Western blotting, qPCR, co-immunoprecipitation, and immunohistochemical staining assays were performed to validate our findings.

**Results:** Alleviated pathological injury and renal function were observed in *Isg15* KO mice with IRI-, cisplatin-, or UUO-induced AKI and the following AKI-to-CKD transition. In hypoxia-reoxygenation, cisplatin or TGF-β1 treated HK-2 cells, knockout ISG15 reduced stimulus-induced cell fibrosis, while overexpression of ISG15 with modification capacity exacerbated cell fibrosis. Immunoprecipitation assays demonstrated that ISG15 promoted ISGylation of TGFβR1, and inhibited its ubiquitination. Moreover, knockout of TGFβR1 blocked ISG15's fibrosis-exacerbating effect in HK-2 cells, while overexpression of TGFβR1 abolished the renal protective effect of ISG15 knockout during IRI-induced kidney injury.

**Conclusions:** ISG15 plays an important role in the development of AKI and subsequent AKI-to-CKD transition by promoting TGFβR1 ISGylation.

## Introduction

Over 10 million individuals worldwide receive new diagnoses of acute kidney injury (AKI), a potentially fatal syndrome characterized by a rapid decline in kidney function, resulting in approximately 1.7 million deaths every year 1, 2. Ischemia-reperfusion injury (IRI), unilateral urethral obstruction (UUO), platinum-based chemotherapeutic agents (cisplatin), contrast agents and other factors are important triggers for AKI 3-8. AKI may develop into irreversible renal fibrosis, which can trigger serious chronic kidney diseases (CKD) 9, 10.

Fibrosis entails the gradual accumulation of extracellular matrix constituents in the kidney, prolonged or extensive fibrosis in AKI contributes to CKD and end-stage renal failure 11, 12. Transforming growth factor-β1 (TGF-β1) is recognized as the most crucial pro-fibrotic factor and the primary signaling pathway driving renal fibrosis 8, 13. TGF-β1 binds and phosphorylates type II TGF-β receptor (TGFβR2), which subsequently phosphorylates the type I TGF-β receptor (TGFβR1) 8, 13. Activation of TGFβR1 then phosphorylates Smad2/3, facilitating their activation and translocation into the nucleus where they bind to relevant co-factors, thereby regulating the transcription of fibrotic genes such as α-smooth muscle actin (α-SMA) and fibronectin 1 (Fn1), thus promoting renal fibrosis 14, 15. On the other hand, activation of TGFβR1 may also trigger Smad-independent signaling, inducing the expression of fibrotic genes 8, 14, 16. Understanding the role of TGFβR1 in this process is essential for developing therapies mitigating fibrosis and preserving renal function in the AKI and the following transition to CKD.

Interferon-stimulated gene 15 (ISG15), an ubiquitin-like protein, initially presents as a 17 kDa precursor protein which undergoes rapid protease-mediated cleavage to yield a mature 15 kDa form (ISG15GG) exposing a C-terminal LRLRGG motif 17. Structurally, ISG15 comprises two β-grasp domains akin to ubiquitin, positioned at its N- and C-terminal, linked by a short hinge region 18. Notably, both the hinge region and the C-terminal LRLRGG residues are indispensable for ISG15 modification 19. ISG15 engages in covalent conjugation with target proteins *via* this LRLRGG motif through a three-step process termed ISGylation 20. Initially, ISG15 is activated by the E1 enzyme UBA7, followed by conjugation facilitated by the E2 enzyme UBCH8, and ultimately ligation mediated by three identified E3 enzymes: HERC5/6, TRIM25, and HHARI. ISGylation is intricately involved in the regulation of various biological processes 19, 21. Concurrently, free ISG15 has been implicated in diverse signaling pathways, including immune regulation 22. Despite its involvement in numerous critical cellular processes, the precise role of ISG15 in AKI and subsequent AKI-to-CKD transition remains elusive.

In this study, *Isg15* knockout (*Isg15* KO) mice were challenged with IRI, cisplatin, or UUO injury. Injured *Isg15* KO mice showed attenuated pathologies and fibrosis, with improved renal function. Additionally, knockout of ISG15 suppressed the cell fibrosis induced by hypoxia-reoxygenation (HR), cisplatin and TGF-β1, while overexpression of ISG15 with conjugation capacity (ISG15GG) has the opposite effect. The Venn analysis of RNA-seq data from three types of AKI animal models, along with *in silico* analysis of protein interactions, suggests a correlation between ISG15 and TGFβR1. Further exogenous and endogenous co-immunoprecipitation (co-IP) assays demonstrated that ISG15 covalently modified TGFβR1 and suppressed its ubiquitination, thereby enhancing the protein stability of TGFβR1. Notably, TGFβR1 overexpression aggregated renal fibrosis *in vivo* and *in vitro*. Moreover, overexpression of TGFβR1 blocked the renal protective effect of ISG15 knockout in IRI-treated mice. Consistently, overexpression of ISG15 failed to promote fibrosis in TGFβR1 knockout HK-2 cells. Together, our results reveal a novel role of ISG15 in regulating renal fibrosis and suggest a potential therapeutic approach against AKI and following AKI-to-CKD transition.

## Materials and Methods

### Animals

Breeding pairs of *Isg15*^+/-^ mice in C57BL/6 background were generated by Cyagen Biosciences (Suzhou, Jiangsu, China). Age-matched male *Isg15* KO mice and wildtype (WT) littermates were used. Genotyping was performed (primers listed in Table S1). Expected Mendelian ratio of mice with different genotypes was found in this study. Male C57BL/6 mice (10-week-old, 25 ± 3 g) were obtained from the Hubei Center for Disease Control and Prevention. Mice were randomly assigned, and housed in a specific-pathogen-free, temperature-controlled (22 ± 1℃) animal facility with a 12-hr light/dark cycle, with free access to water/food. Animals were handled according to the Guidelines of the China Animal Welfare Legislation, as approved by the Committee on Ethics in the Care and Use of Laboratory Animals, College of Life Sciences, Wuhan University (WDSKY0201705-2, Wuhan, China).

### AKI models and treatments

Ischemic AKI was induced using both bilateral IRI (BIRI) and unilateral renal IRI (UIRI) models. For the BIRI model, mouse was anesthetized and underwent midline abdominal incisions, both kidneys were clamped to block blood flow for 22 minutes. After ischemia, clamps were released to start reperfusion. For the UIRI model was used as we previously described 23, 24, left renal pedicle of mouse was bluntly clamped for 30 minutes, reperfusion was achieved by removing the clamp. Sham operated mice (Sham) were used as respective controls. Mice were sacrificed at day 1, 2 or 3 after BIRI; or at day 1, 3, 7 and 21 after UIRI. Death of some animals were observed at Day 3 of BIRI, but not during the UIRI study. For cisplatin-induced AKI, a single dose of 30 mg/kg body-weight cisplatin (T1564, Targetmol, Shanghai, China) was injected intraperitoneally and the mice were euthanized at Day 3 after the injection 25. For the UUO model, the left ureter of the mouse was ligated at proximal and distal points and then cut between the ligated points 26. Mice were sacrificed 7 days later and kidneys were collected.

### Intrarenal adenovirus delivery

TGFβR1 overexpression adenovirus and control adenovirus (Genechem, Shanghai, China) were delivered into the mouse kidney by renal intraparenchymal injection, followed by IRI modeling experiments. Initially, the mice were temporarily anesthetized, and their abdominal cavity was opened to expose the unilateral kidneys. Then, 150 μL of TGFβR1 or control adenovirus (6 × 10^10^ pfu/mL) was aspirated with a 31G needle. Four to six sites were selected from each kidney the slow injection of the virus into the renal cortex, as previously described 27.

### Assessment of renal function

Renal function was evaluated by measuring serum creatinine (CREA) and blood urea nitrogen (BUN) levels using a creatinine reagent kit and a BUN reagent kit 28, respectively (both from Jiancheng Bio., Nanjing, China).

### Histological and immunohistochemical studies

Paraffin-embedded sections were deparaffinized and rehydrated as previously reported 29, 30. For pathological evaluation, H&E stained renal sections were assessed and evaluated as previously described 29, 30. For evaluation of fibrosis, a Masson staining kit (Solarbio, Wuhan, China) was used. For immunohistochemical staining, primary antibodies for α-SMA, Fn1 (information listed in Table S2) were applied overnight at 4 °C. Sections were then incubated with respective biotinylated secondary antibody, followed by incubation in ABC-peroxidase solution (Vector laboratories, Burlingame, CA), and visualized using 3,3′-diaminobenzidine (DAB, CWbiotech, Beijing, China). Quantitative analysis of positively stained cells was performed as previously reported 29, 30. For immunofluorescent staining, tissue sections and cryosections were incubated overnight with respective primary antibodies (information listed in Table S2). After washing, sections were incubated with respective Alexa Fluor secondary antibody (Thermo Fisher, Waltham, MA). Sections were covered with DAPI and anti-fading medium. Images were taken by a confocal microscope (Nikon AX2, Japan).

### Cell culture

Human renal tubular cell line HK-2 (GDC0152, from CCTCC, China Center for Type Culture Collection, Wuhan, China) was cultured in DMEM/F12 media (Hyclone, Palo Alto, CA) plus 10% FBS. HEK293T cells (CL-0005, Procell Biotech) were cultured in DMEM media (Hyclone) containing 10% FBS. HK-2 and HEK293T cells were analyzed with authenticated STR locus and tested for mycoplasma contamination, by CCTCC or Procell Biotech. *In vitro* HR experiments were performed as previously described with slight modifications 31. At 80% confluence, HK-2 cells were placed in a 37 ℃ incubator under 1% O_2_ with the regular media replaced by the glucose- and FBS-free media to mimic hypoxia. After 24-hr culturing under the hypoxia condition, HK-2 cells were returned to the regular culture condition for another hour to simulate reperfusion. For cisplatin treatments, HK-2 cells were treated with 5 μg/mL cisplatin for 24 hrs. For TGF-β1 (Peprotech, 100-21-10) treatment, HK-2 cells were treated with 10 ng/mL TGF-β1 for 24 hrs.

### Plasmids and transfection

Plasmids encoding human ISG15GG (myc-ISG15GG), a mature form capable of ISGylation, and ISG15AA (myc-ISG15AA), which lacks ISGylation activity and represents free ISG15, were generously provided by Dr. Jin-Hyun Ahn (University of Erlangen-Nuremberg). The CRISPR-Cas9-based protocols for genome engineering were used as described 32. CRISPR-Cas9 vectors containing gRNA targeting ISG15 (5'-ATCTGCCTTACCATGGCTG-3'), TGFβR1 (5'-CACCGCATACAAACGGCCTATCTCG-3'), were used to knockout ISG15, TGFβR1, respectively. Expression plasmids for HA-tagged E1, E2, E3 and Flag-tagged TGFβR1 were constructed (information listed in Table S1). Most cells were transfected with indicated plasmids for six hours, then treated with/without HR, cisplatin, TGF-β1 before collection.

### Quantitative real-time PCR (qPCR)

qPCR was performed as we previously described 33. Total RNA was isolated from the kidneys or cultured cells using RNA^iso^ Plus (TaKaRa Biotech, Dalian, China). Total RNA was reverse transcribed into cDNA using an M-MLV first strand synthesis system (Invitrogen, Grand Island, NY). The abundance of specific gene transcripts was assessed by qPCR. The mRNA levels of specific genes were normalized to β-actin or Rn18s. Primers used are listed in Table S3.

### Western blots

Freshly collected kidney or cultured cells were sonicated in ice-cold RIPA buffer (Beyotime), and protein concentrations were quantitated as we previously described 34, 35. 20-80 μg protein from each sample was separated by SDS-PAGE. The proteins were transferred onto PVDF membranes for immune detection. Antibodies used are shown in Table S2.

### Co-Immunoprecipitation (Co-IP)

Cells were lysed with pre-lysis buffer (25 mM Tris-HCl, pH 7.4, 150 mM NaCl, 1% NP-40, 1 mM EDTA, 5% glycerol), cell lysate was sonicated and centrifuged at 12000 g for 10 min. Then cell lysates were incubated with the indicated antibodies (Table S2) or respective IgGs with Dynabeads Protein G (Thermo Scientific, Waltham, MA) overnight at 4 °C. After washing (pre-lysis buffer with additional 50 mM NaCl), the beads were boiled in loading buffer and subjected to immunoblotting 36, 37.

### RNA sequencing (RNA-seq) and analysis

Total renal RNA was isolated from IRI-treated mice for RNA-seq, and the data has been uploaded to the NCBI Gene Expression Omnibus database (GSE183455) 33. And published RNA-seq data deposited in NCBI GEO database (GSE216376 and GSE207587) were retrieved, and analyzed *via* GEO integrated analyzing tool (GEO2R) or R3.5.2 for the heatmap. Log2 fold change of differentially expressed genes and ISG15 were visualized.

### Protein-Protein Interaction (PPI) Modeling of ISG15 and TGFβR1

ISG15- TGFβR1 interaction was modeled using Alphafold 2. Query sequence and multiple sequence alignment (MSA) from MMseqs2 served as input without templates 38. MSA generation and AlphaFold 2 predictions were performed using ColabFold (https://colab.research.google.com/ssssub/sokrypton/ColabFold). Modeling results were analyzed using PDBePISA (www.ebi.ac.uk/msd-srv/prot_int/pistart.html). The binding strength of ISG15 and TGFβR1, TGFβR2, were analyzed by Δ^i^G (solvation energies) and root-mean-square deviation (RMSD).

### Single cell RNA-seq (scRNA-seq) data analysis

The scRNA-seq data of human kidney allograft biopsy and mice IRI were obtained respectively from GSE109564 and GSE139107, which contain the gene expression matrix data and cell cluster annotations. Then, we performed data normalization and scaling according to standard pre-processing workflow of the Seurat (version V5, https://satijalab.org/seurat/).

### Statistics

Data were analyzed with GraphPad Prism (version 8). Statistical analysis was performed using two-tailed Student's t test for two experimental groups, and one-way ANOVA for multiple experimental groups without adjustment. Data are reported as the mean values with error bars showing the standard deviation (SD). For cell culture experiments, at least three independent experiments were performed with similar results. For animal study, the n number of biological independent animals in different groups are provided in figure legends. A *p* value of less than 0.05 was considered statistically significant.

## Results

### ISG15 is upregulated during renal IRI-induced kidney injury

**Figure 1:**
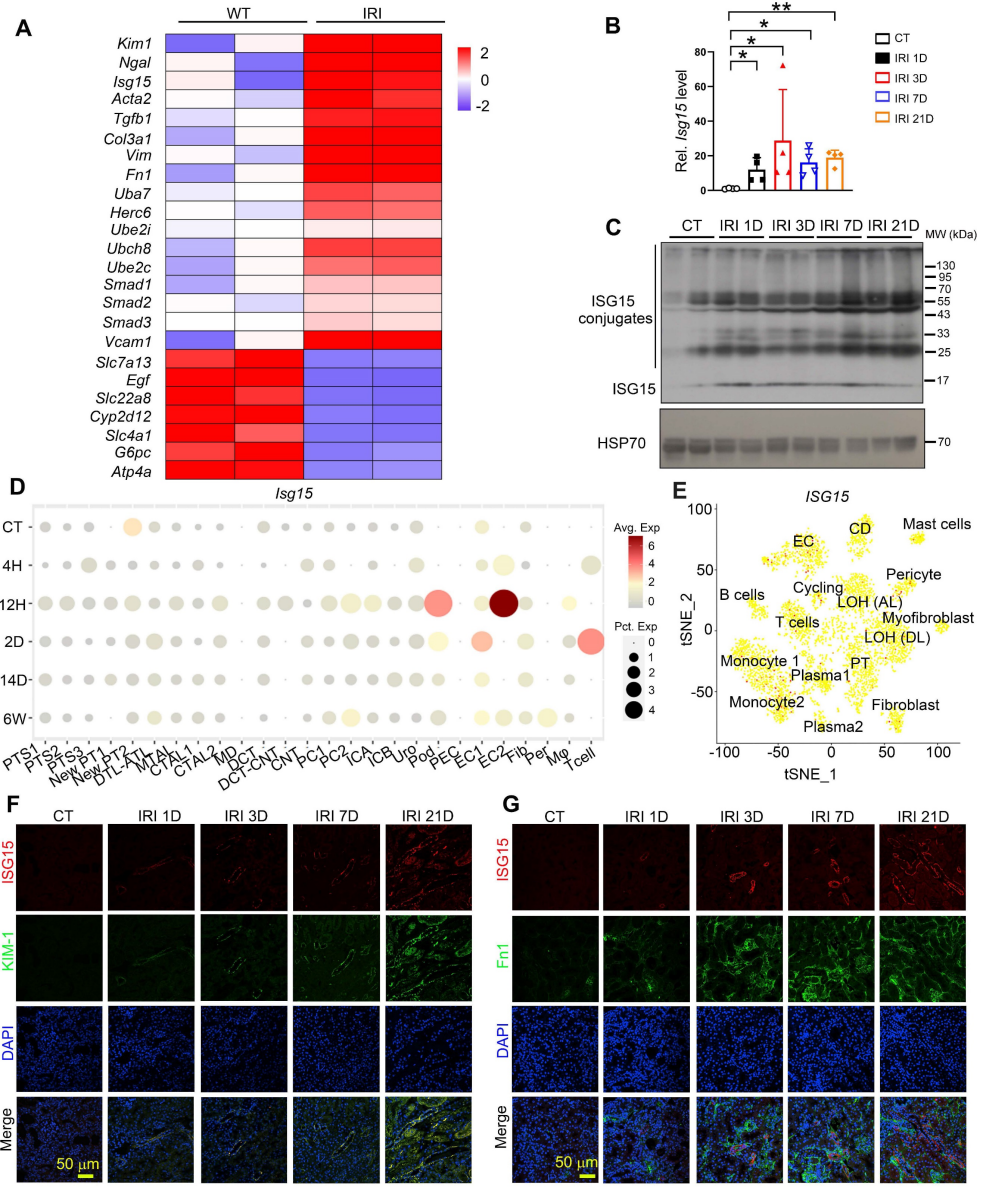
** ISG15 is up-regulated in the kidney of mice after renal IR injury. (A)** RNA-seq data of ISG15 and its related ligase genes, and fibrotic genes in the kidneys of IRI-treated mice. **(B)** qPCR results of *Isg15* of non-injured (CT) or IRI-treated mice at 1, 3, 7, or 21 days, n = 4 per group, *P < 0.05; **P < 0.01. **(C)** Western blot of ISG15 at indicated times after IRI, n = 2 per group. **(D)** single cell RNA-seq data of ISG15 in the kidneys of IRI mice. CT, control; 4H, 4 hours; 12H, 12 hours; 2D, 2 days; 14D, 14 days; 6W, 6 weeks. **(E)** tSNE plot of ISG15 expressed cell clusters from single cell RNA-seq of a human kidney allograft biopsy. CD, collecting duct; EC, endothelial cell; LOH (AL), loop of Henle, ascending limb; LOH (DL), loop of Henle, distal limb; PT, proximal tubule. **(F)** Representative images of ISG15 (red), KIM-1 (green) and DAPI (blue) staining of the kidney. **(G)** Representative images of ISG15 (red), Fn1 (green) and DAPI (blue) staining of the kidney. CT, non-injured mice; IRI 1D/3D/7D/21D, mice at IRI 1/3/7/21 days, n = 4 per group. Scale bar = 50 μm.

To investigate the role of ISG15 in IRI-induced kidney injury, we analyzed our previous RNA-seq data of IRI model, and found that *Isg15* and its E1/E2/E3 ligases levels were increased in IRI injured kidneys (Figure 1A). Further validation through qPCR and Western blots confirmed that the gene and protein levels of ISG15 increased at different time points following IRI (Figure 1B-C). Moreover, to explore the cellular sources of ISG15 in IRI injured kidney, we analyzed published scRNA-seq data of a human kidney allograft biopsy and a mouse IRI model (Figure 1D-E). ISG15 was ubiquitously expressed in a variety of cells. However, with prolonged injury duration, sustained high expression of ISG15 is observed in the proximal tubules and fibroblasts (Figure 1D-E). Co-staining of ISG15 with KIM1 or Fn1 consistently demonstrated weak ISG15 staining in tubular cells and fibroblast under non-injury conditions; after IRI, a significantly increased ISG15 level was observed in renal tubules and fibroblast of IRI mice (Figure 1F-G).

### Knockout of ISG15 attenuates renal IRI induced kidney injury

To study the role of ISG15 in renal IRI, *Isg15* knockout (*Isg15* KO; *Isg15^-/-^*) mice and their age-matched wildtype (WT; *Isg15*^+/+^) littermates were studied (Figure S1A-B). The deletion efficiency of ISG15 in the kidney was examined, ISG15 gene and protein levels were barely detectable in the kidneys of *Isg15* KO mice (Figure S1C-D).

**Figure 2:**
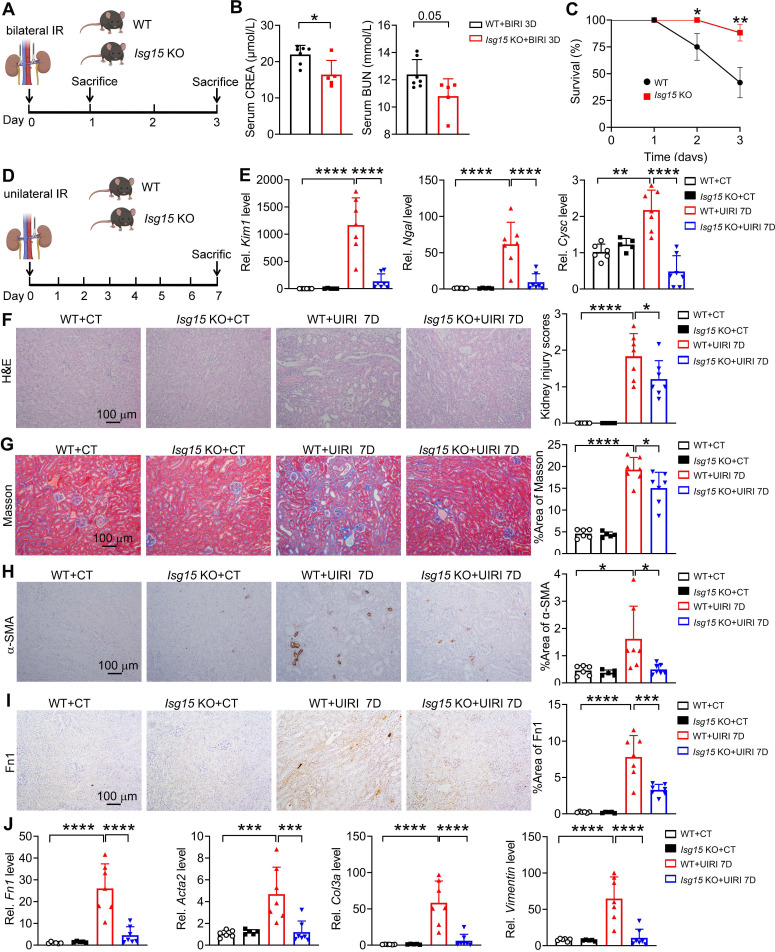
** Knockout of ISG15 suppresses IRI-induced kidney injury. (A)** Experimental design chart of bilateral IRI (BIRI). **(B)** Serum creatinine (left) and BUN (right) levels for indicated experimental groups at 3 days after BIRI 3D. WT+BIRI 3D, n = 7; *Isg15* KO+BIRI 3D, n = 5. **(C)** Survival rate of WT and *Isg15* KO mice were subjected to BIRI. WT+BIRI, n = 12; *Isg15* KO+BIRI, n = 14.** (D)** Experimental design chart of unilateral IRI (UIRI). **(E)** qPCR results of *Kim1*, *Ngal*,* Cysc* for WT and *Isg15* KO mice at non-injured conditions (CT) or at 7 days after UIRI. **(F)** Representative H&E images (left) with injury scores (right) of the kidney of WT and *Isg15* KO mice at non-injured conditions (CT) or at 7 days after UIRI. Scale bar = 100 μm.** (G-I)** Representative images and quantitative results of Masson staining (G), immunostaining for α-SMA (H), Fn1 (I) of the kidney of WT and *Isg15* KO mice at non-injured conditions (CT) or at 7 days after UIRI. Scale bar = 100 μm. **(J)** qPCR results of* Fn1*, *Acta2*,* Col3a* and* Vimentin* in the kidney of WT and *Isg15* KO mice at non-injured conditions (CT) or at 7 days after UIRI. WT+CT, n = 6; *Isg15* KO+CT, n = 5; WT+UIRI 7D, n = 7; *Isg15* KO+UIRI 7D, n = 7. *P < 0.05; **P < 0.01; ***P < 0.001; ****P < 0.0001.

In order to gain a detailed understanding of the specific role of ISG15 in AKI and following AKI-to-CKD transition, we established multiple kidney injury models and collected samples at different time points. At first, knockout of ISG15 did not affect renal function in mice subjected to bilateral IRI (BIRI) at day 1 (Figure S2); however, at day 3 post-BIRI, ISG15 knockout significantly improved renal function and reduced mortality caused by BIRI (Figure 2A-C). Due to the high mortality rate observed in mice subjected to BIRI, we established unilateral IRI (UIRI) models for 7 days (Figure 2D). ISG15 knockout significantly reduced kidney damage in mice at 7 days post-UIRI (Figure 2E-F). Fibrosis markers, such as collagen deposition (Masson staining), α-smooth muscle actin (α-SMA, gene* Acta2*) and Fibronectin (Fn1) were analyzed, while *Isg15* KO markedly decreased the collagen deposition, and levels of α-SMA and Fn1 after renal UIRI (Figure 2G-I). Additionally, the transcriptional levels of ISGylation related E1/E2/E3 ligases (UBA7, UBCH8, HERC6) were also increased in the kidneys of UIRI-injured mice (Figure S3A). And, the mRNA levels of fibrotic genes (*Fn1*,* Acta2*, *Col3a, Vimentin*) were markedly elevated after renal UIRI (Figure 2J); *Isg15* KO significantly inhibited the transcriptional levels of *Fn1*, *Acta2*, *Col3a, Vimentin* (Figure 2J).

### Knockout of ISG15 improves UUO- or cisplatin- induced kidney injury

**Figure 3:**
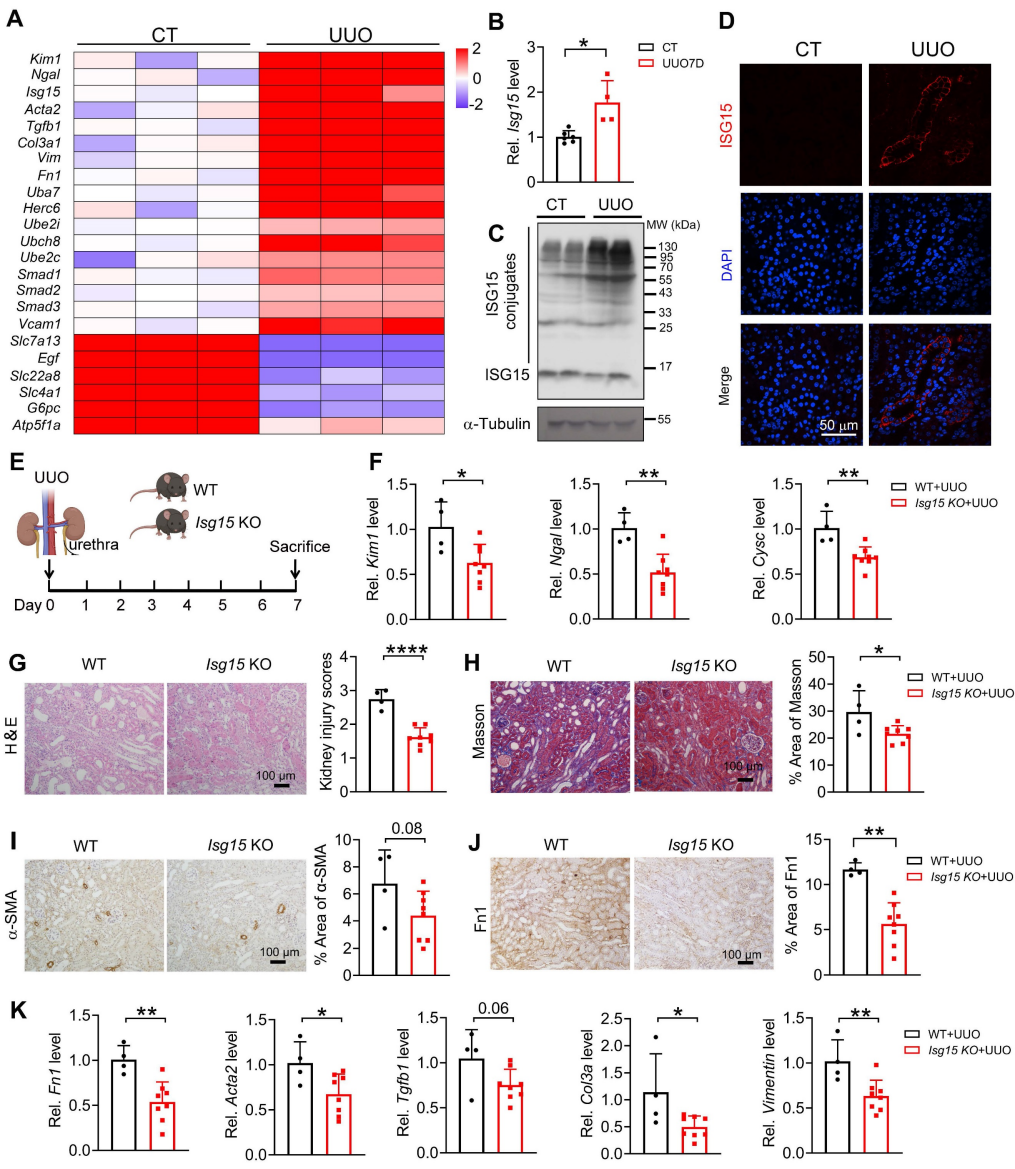
** Knockout of ISG15 suppresses UUO-induced kidney injury. (A)** RNA-seq data of ISG15 and its related ligase genes, and fibrotic genes in the kidneys of UUO injured mice. **(B-C)** qPCR (B) and WB (C) results of ISG15 of non-injured (CT) or UUO injured mice. **(D)** Representative images of ISG15 (red), and DAPI (blue) staining of the kidney of CT or UUO injured mice. CT, n = 6; UUO, n = 5.** (E)** Experimental design chart of UUO injury. **(F)** qPCR results of *Kim1*, *Cysc*, *Ngal* in the kidneys of WT and *Isg15* KO mice after UUO injury. **(G)** Representative H&E images (left) with injury scores (right) of the kidney of WT and *Isg15* KO mice after UUO injury. Scale bar = 100 μm.** (H-J)** Representative images and quantitative results of Masson staining (H), immunostaining for α-SMA (I), Fn1 (J) of the kidney of WT and *Isg15* KO mice after UUO injury. Scale bar = 100 μm. **(K)** qPCR results of *Fn1*, *Tgfb1*,* Acta2*,* Col3a* and* Vimentin* in the kidney of WT and *Isg15* KO mice after UUO injury. WT+UUO, n = 4; *Isg15* KO+UUO, n = 8. *P < 0.05; **P < 0.01.

To investigate whether ISG15 also responds in other renal injury models, UUO- or cisplatin-induced kidney injury model was applied. Similar to IRI model, we analyzed published RNA-seq data of UUO injured kidney, *Isg15* and its related E1/E2/E3 ligases levels were significantly increased after UUO injury (Figure 3A). Consistent with the RNA-seq data, ISG15 transcriptional and protein levels, as well as its related E1/E2/E3 ligases levels were upregulated in the tubular of UUO injured kidneys (Figure 3B-D and Figure S3B). *Isg15* KO consistently decreased the mRNA levels of the kidney injury markers *Kim1*, *Ngal*, *Cysc*, and inhibited renal pathological injury in the UUO injured mice (Figure 3E-G). Masson, α-SMA and Fn1 staining showed reduced fibrosis in *Isg15* KO mice compared to WT mice after UUO injury (Figure 3H-J). Meanwhile, the mRNA levels of fibrotic factors *Fn1*, *Acta2*, *Col3a, Vimentin* were decreased in UUO-injured *Isg15* KO mice (Figure 3K).

Meantime, in published RNA-seq data of cisplatin-injured kidney, *Isg15* and its related E1/E2/E3 ligases, TGF-β1 signaling gene levels were increased at day 3 post cisplatin injection (Figure S4A). Consistently, ISG15 transcriptional and protein levels were upregulated in the tubular of cisplatin-injured kidneys (Figure S4B-D). Knockout of* Isg15* decreased serum creatinine and BUN levels, improved tubular injury, reduced fibrosis in cisplatin-injured mice (Figure S4E-L).

### ISG15 exacerbates HR induced renal fibrosis in cultured renal tubular cells

**Figure 4:**
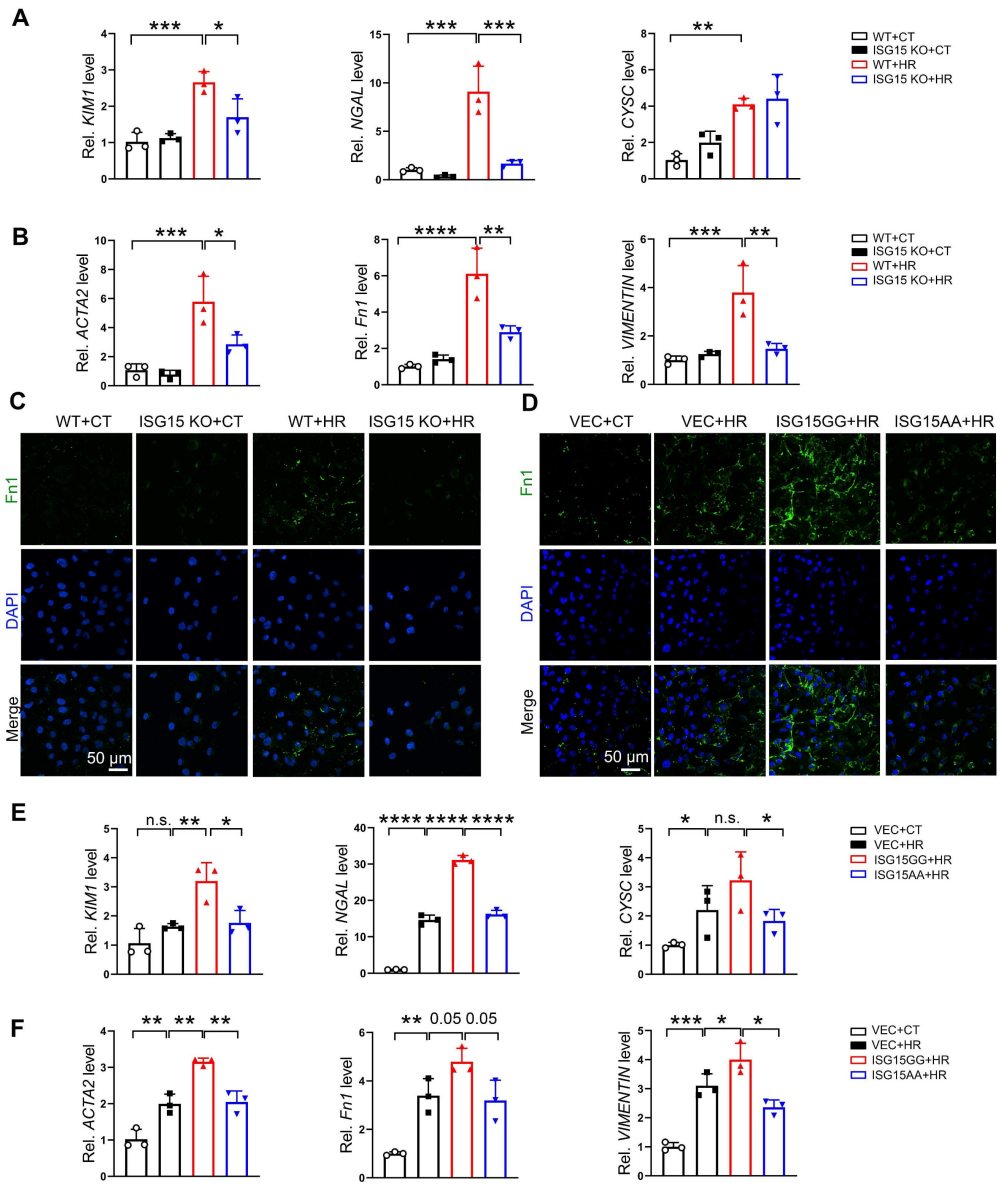
** ISG15 aggravates HR induced fibrosis in cultured HK-2 cells. (A-B)** qPCR results of *KIM1*, *NGAL, CYSC*,* ACTA2*,* Fn1* and* VIMENTIN* in ISG15 knockout HK-2 cells with or without HR treatment. **(C)** Representative Fn1 images in ISG15 knockout HK-2 cells with or without HR treatment. **(D)** Representative Fn1 images in ISG15 overexpressed HK-2 cells with HR treatment. Scale bar = 50 μm.** (E-F)** qPCR results of *KIM1*, *NGAL, CYSC*,* ACTA2*,* Fn1* and* VIMENTIN* in ISG15 overexpressed HK-2 cells with HR treatment. The experiments were repeated three times, and at least three biological replicates per group were used. *P < 0.05; **P < 0.01; ***P < 0.001; ****P < 0.0001; n.s., not significant.

To clarify the role of ISG15 in the AKI and AKI-to-CKD transition, we investigated the effects of ISG15 on HR-injured cells *in vitro*. ISG15 knockout or overexpressed HK-2 cells were generated (Figure S5A-B). The mRNA levels of the kidney injury and fibrotic factors, as well as Fn1 protein level, were up-regulated in HR treated cells, while knockout of ISG15 suppressed the *KIM1*, *NGAL*, *ACTA2, Fn1, VIMENTIN*, and Fn1 protein level in HR-treated HK-2 cells (Figure 4A-C). Notably, in HR-treated HK-2 cells, overexpression of ISG15GG (mature form with ISGylation capability) aggravated HR induced cell fibrosis, while overexpression of ISG15AA (lacking ISGylation ability and represents free ISG15) showed no obvious effect (Figure 4D-F).

### ISG15 aggravates TGF-β1 or cisplatin induced renal fibrosis in renal tubular epithelial cells

**Figure 5:**
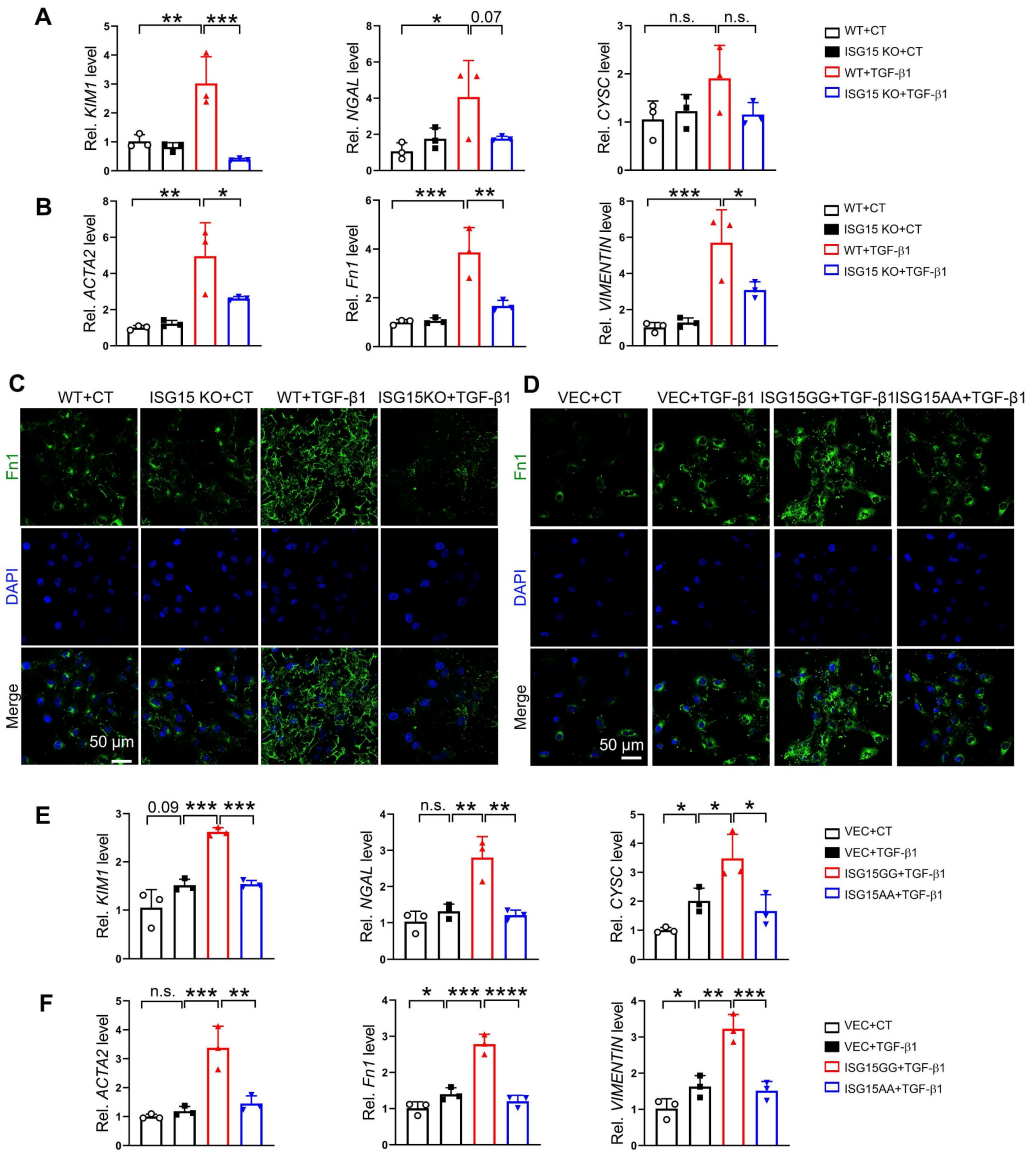
** ISG15 aggravates TGF-β1 induced fibrosis in cultured HK-2 cells. (A-B)** qPCR results of *KIM1*, *NGAL, CYSC*,* ACTA2*,* Fn1* and* VIMENTIN* in ISG15 knockout HK-2 cells with or without TGF-β1 treatment. **(C)** Representative Fn1 images in ISG15 knockout HK-2 cells with or without TGF-β1 treatment. **(D)** Representative Fn1 images in ISG15 overexpressed HK-2 cells with TGF-β1 treatment. Scale bar = 50 μm.** (E-F)** qPCR results of *KIM1*, *NGAL, CYSC*,* ACTA2*,* Fn1* and* VIMENTIN* in ISG15 overexpressed HK-2 cells with TGF-β1 treatment. The experiments were repeated three times, and at least three biological replicates per group were used. *P < 0.05; **P < 0.01; ***P < 0.001; ****P < 0.0001; n.s., not significant.

The effects of ISG15 on renal cell fibrosis was further examined in HK-2 cells treated with TGF-β1, a classic fibrosis inducer. TGF-β1 induced up-regulation of *KIM1*, *NGAL, ACTA2, Fn1, VIMENTIN* mRNA levels, as well as Fn1 protein level, which was inhibited by ISG15 knockout (Figure 5A-C). As expected, overexpression of ISG15GG aggravated TGF-β1 induced renal fibrosis, while overexpression of ISG15AA showed no obvious effect on fibrosis (Figure 5D-F). Similarly, ISG15 knockout inhibited cisplatin-induced cell damage, whereas ISG15GG overexpression exacerbates cisplatin-induced cell damage (Figure S6).

### ISG15 ISGylates TGFβR1 in renal tubular epithelial cells

**Figure 6:**
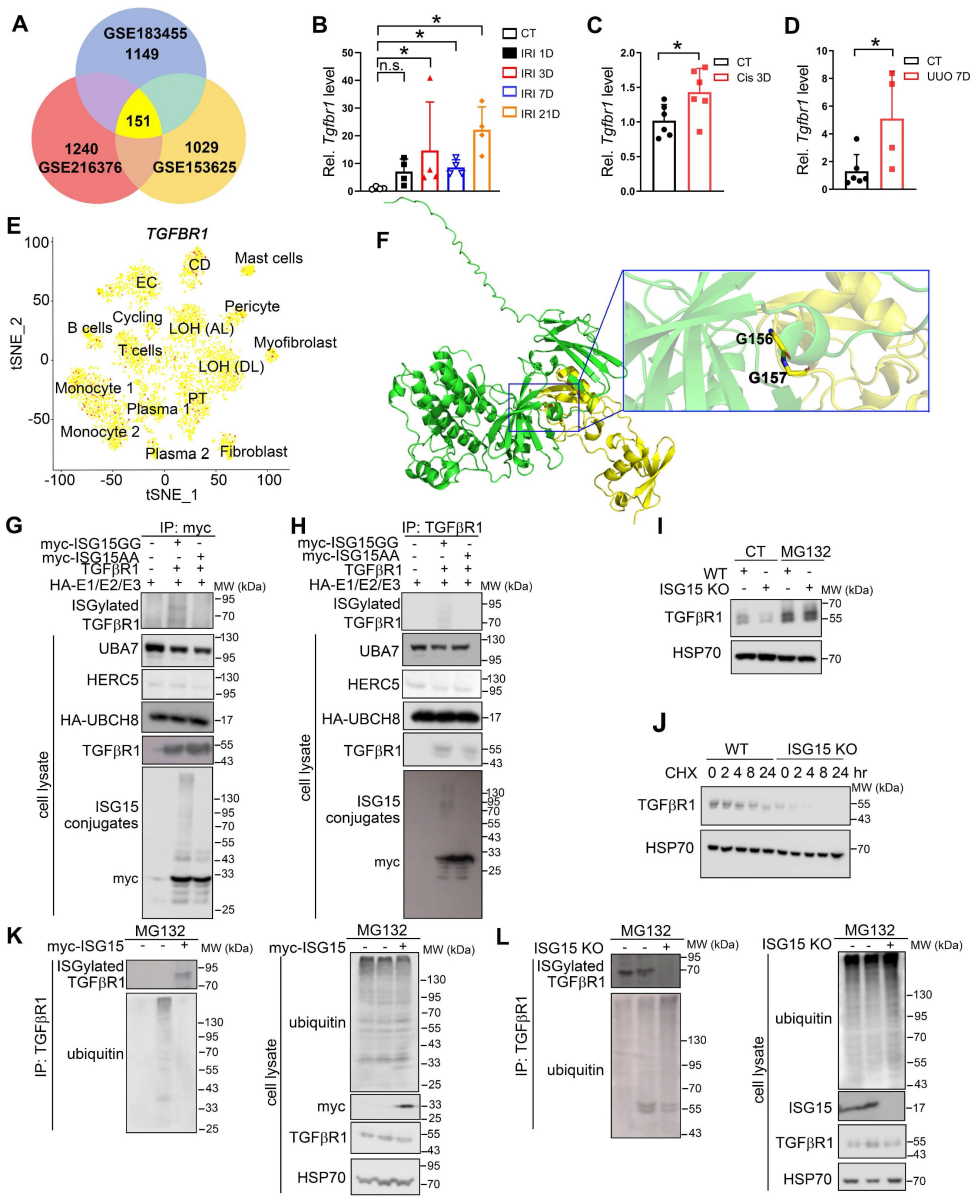
** ISG15 binds and ISGylates TGFβR1 receptor. (A)** Venn diagram analysis of three RNA-seq data. **(B-D)** qPCR results of *Tgfbr1* in the kidneys after IRI (B), cisplatin (C), UUO (D) injury at indicated time, n = 4-6 per group. *P < 0.05; n.s., not significant. **(E)** tSNE plot of TGFβR1 expressed cell clusters from scRNA-seq of a human kidney allograft biopsy. CD, collecting duct; EC, endothelial cell; LOH (AL), loop of Henle, ascending limb; LOH (DL), loop of Henle, distal limb; PT, proximal tubule. **(F)** Binding model of ISG15 and TGFβR1.** (G-H)** Co-IP results of ISG15 and TGFβR1 in HEK293T cells transfected with indicated plasmids. **(I)** Effects of MG132 on ISG15 knockout-mediated proteolysis of TGFβR1. The cells were treated with MG132 (10 μM) for 6 h before immunoblots.** (J)** Effects of ISG15 knockout on the protein levels of TGFβR1 under the treatment of a ribosome inhibitor CHX (100 μM) for 6 h before immunoblots. **(K)** ISG15 overexpression reduced ubiquitination of endogenous TGFβR1 in HEK293T cells treated with MG132. HSP70 serves as the loading control. **(L)** ISG15 knockout enhanced ubiquitination of endogenous TGFβR1 in HK-2 cells treated with MG132. HSP70 serves as the loading control. The experiments were repeated three times.

To uncover the downstream effectors of ISG15, we analyzed the RNA-seq data of IRI-, cisplatin-, or UUO models to extract upregulated genes. Subsequently, we identified a crucial receptor in the fibrosis pathway, TGFβR1 (Figure 6A), which may suggest a potential interaction between ISG15 and TGFβR1. *Tgfbr1* transcriptional levels were upregulated in IRI-, cisplatin-, or UUO-injured kidneys (Figure 6B-D). Moreover, scRNA-seq data revealed that TGFβR1 was ubiquitously expressed in a variety of cells (Figure 6E), which was similar to ISG15 distribution. Leveraging AlphaFold2 predictions, we found evidence of a possible interaction between ISG15 and TGFβR1 (Δ^i^G = -7.3 kcal/mol, with Δ^i^G < -2 kcal/mol indicating interaction, Figure 6F and Table S4). We further confirmed the ISG15-TGFβR1 interaction by co-IP assays. Exogenous co-IP experiments demonstrated that ISG15 covalently conjugated TGFβR1 (Figure 6G-H). Additionally, overexpression of USP18, a de-ISGylation enzyme, decreased ISGylation level of TGFβR1 (Figure S7).

Notably, decreased TGFβR1 and p-Smad2 levels were observed in the *Isg15* KO mice injured with IRI, cisplatin, or UUO injury (Figure S8). Treatments with proteasome inhibitor (MG132) and protein synthesis inhibitor (cycloheximide, CHX) were performed to examine whether ISG15 affect the stability of TGFβR1. MG132 completely blocked ISG15 knockout-mediated TGFβR1 degradation, whereas CHX showed no effect (Figure 6I-J). To address whether ISG15 regulates TGFβR1 stability by ubiquitination, ubiquitination assays were performed. Overexpression of ISG15 enhanced TGFβR1 ISGylation, and inhibited ubiquitination of TGFβR1 (Figure 6K), while ISG15 knockout showed the opposite effects (Figure 6L).

### TGFβR1 aggregates AKI and subsequent AKI-to-CKD transition *in vitro* and *in vivo*

**Figure 7:**
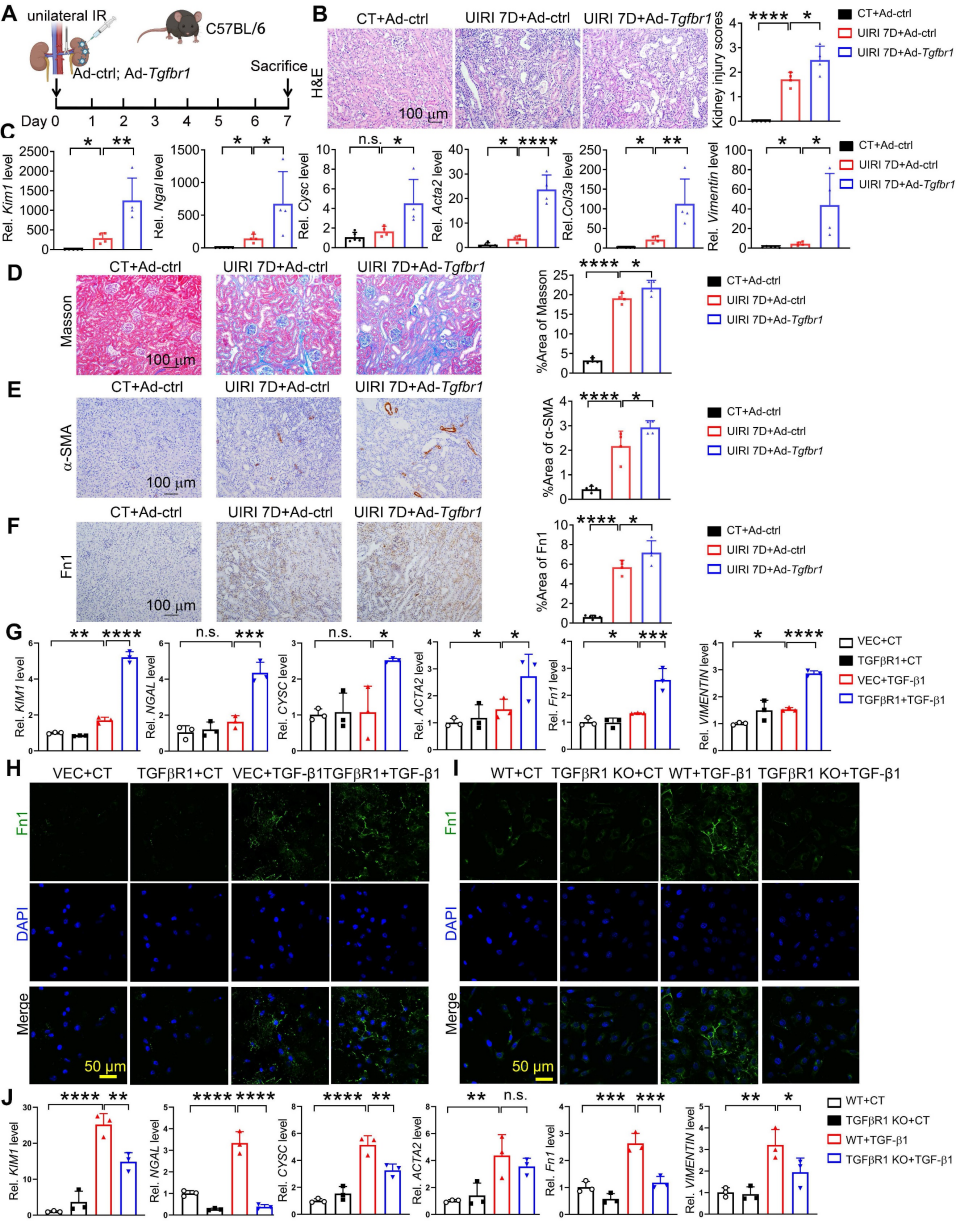
** TGFβR1 exacerbates AKI and the following AKI-to-CKD transition *in vivo* and *in vitro*. (A)** Experimental design chart of TGFβR1 overexpression by renal intraparenchymal injection in UIRI-treated mice. **(B)** Representative H&E images (left) with injury scores (right) of the kidney of indicated groups. Scale bar = 100 μm.** (C)** qPCR results of *Kim1*, *Ngal*,* Cysc*, *Acta2*,* Fn1*,* Col3a* and* Vimentin* in the kidney of indicated groups. **(D-F)** Representative images and quantitative results of Masson staining (D), immunostaining for α-SMA (E), Fn1 (F) of the kidney of indicated groups. Scale bar = 100 μm. CT+Ad-ctrl, n = 5; UIRI+Ad-ctrl, n = 4; UIRI+Ad-*Tgfbr1*, n = 4. **(G)** qPCR results of *KIM1*, *NGAL, CYSC*,* ACTA2*,* Fn1* and* VIMENTIN* in TGFβR1 knockout HK-2 cells with or without TGF-β1 treatment. **(H)** Representative Fn1 images in TGFβR1 overexpressed HK-2 cells with or without TGF-β1 treatment. Scale bar = 50 μm.** (I)** Representative Fn1 images in TGFβR1 overexpressed HK-2 cells with or without TGF-β1 treatment. Scale bar = 50 μm.** (J)** qPCR results of *KIM1*, *NGAL, CYSC*,* ACTA2*,* Fn1* and* VIMENTIN* in TGFβR1 knockout HK-2 cells with or without TGF-β1 treatment. The experiments were repeated three times, and at least three biological replicates per group were used. *P < 0.05; **P < 0.01; ***P < 0.001; ****P < 0.0001; n.s., not significant.

To verify the effect of TGFβR1 in AKI and subsequent AKI-to-CKD transition, we constructed a TGFβR1 overexpression animal model by renal intraparenchymal injection of TGFβR1 overexpressing adenovirus (Figure S9). Overexpression of TGFβR1 exacerbated renal pathological injury, and increased mRNA levels of *Kim1*, *Ngal*, *Cysc*, *Acta2*, *Col3a*, *Vimentin* in the kidneys of mice subjected to UIRI (Figure 7A-C). Additionally, overexpression of TGFβR1 aggravated the renal fibrosis in the kidney of mice after UIRI 7D (Figure 7D-F). *In vitro*, overexpression of TGFβR1 led to increased transcriptional levels of* KIM1*, *NAGL*, *CYSC*, *ACTA2*, *Fn1*, and *VIMENTIN*, along with elevated Fn1 protein level in TGF-β1 treated HK-2 cells (Figure 7G-H). Additionally, knockout of TGFβR1 reduced renal cell injury and fibrosis in TGF-β1 treated HK-2 cells (Figure 7I-J).

### ISG15 accelerates AKI and subsequent AKI-to-CKD transition by ISGylating TGFβR1

**Figure 8:**
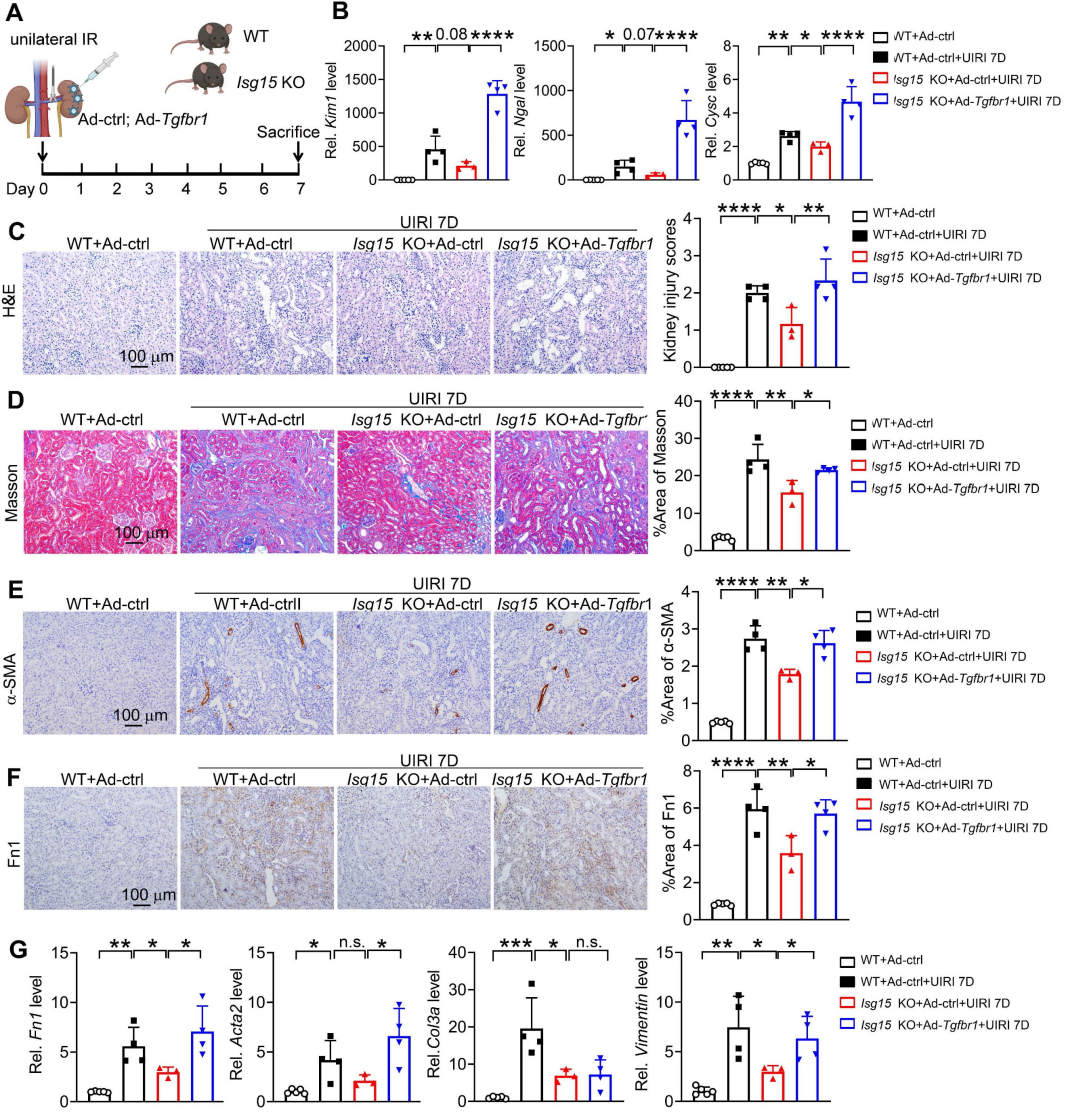
** Renal overexpression of TGFβR1 abolishes the renal protective effect of ISG15 knockout. (A)** Experimental design chart of TGFβR1 overexpression by renal intraparenchymal injection in *Isg15* KO mice subjected to UIRI. **(B)** qPCR results of *Kim1*, *Ngal*,* Cysc* in the kidney of indicated groups.** (C)** Representative H&E images (left) with injury scores (right) of the kidney of different groups. Scale bar = 100 μm.** (D-F)** Representative images and quantitative results of Masson staining (D), immunostaining for α-SMA (E), Fn1 (F) of the kidney of indicated groups. Scale bar = 100 μm. **(G)** qPCR results of *Fn1*, *Acta2*,* Col3a* and* Vimentin* in the kidney of different groups. WT+Ad-ctrl, n = 5; WT+UIRI+Ad-ctrl, n = 4; *Isg15* KO+UIRI+Ad-ctrl, n = 3; *Isg15* KO+UIRI+Ad-*Tgfbr1*, n = 4. *P < 0.05; **P < 0.01; ***P < 0.001; ****P < 0.0001; n.s., not significant.

**Figure 9:**
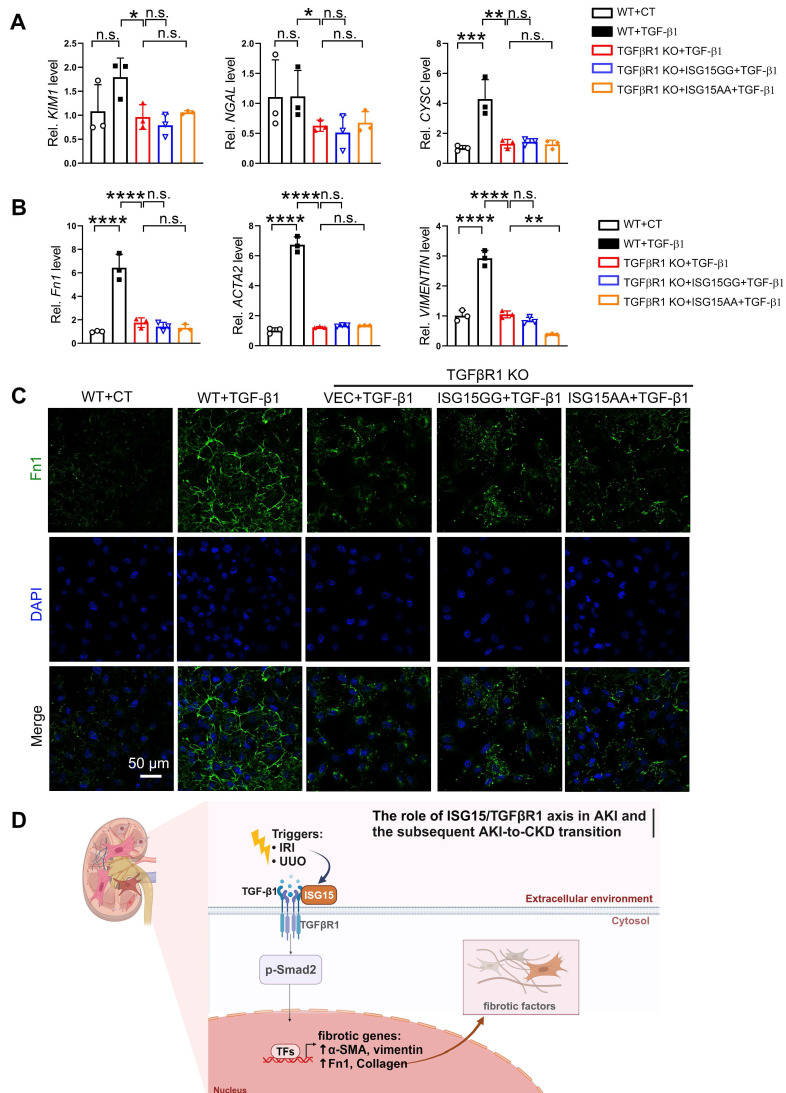
** ISG15 aggravates renal injury by ISGylating TGFβR1. (A)** qPCR results of *KIM1*, *NGAL, CYSC* in TGFβR1 knockout HK-2 cells with ISG15 overexpression under TGF-β1 treatment. **(B)** qPCR results of *ACTA2*,* Fn1* and* VIMENTIN* in TGFβR1 knockout HK-2 cells with ISG15 overexpression under TGF-β1 treatment.** (C)** Representative Fn1 images in TGFβR1 knockout HK-2 cells with ISG15 overexpression under TGF-β1 treatment. Scale bar = 50 μm.** (D)** A schematic model of the ISG15 role involved in AKI and the following AKI-to-CKD transition. The experiments were repeated three times, and at least three biological replicates per group were used. *P < 0.05; **P < 0.01; ***P < 0.001; ****P < 0.0001; n.s., not significant.

To confirm whether TGFβR1 is involved in the role of ISG15 in AKI and its transition to CKD, we overexpressed TGFβR1 in the kidneys of *Isg15* KO mice. Compared with wildtype mice treated with UIRI, *Isg15* KO mice showed reduced levels of the kidney injury markers *Kim1*, *Ngal*, *Cysc* along with less renal pathological injury, which was abolished by TGFβR1 overexpression (Figure 8A-C). Similarly, the renal fibrotic-suppressive effect of *Isg15* KO was counteracted by overexpression of TGFβR1 (Figure 8D-G). *In vitro*, TGF-β1 stimulation led to an increase in the transcriptional levels of *CYSC*, *ACTA2*, *Fn1*, and *VIMENTIN*, along with an elevation in Fn1 protein level, which was suppressed by knockout of TGFβR1 (Figure 9A-C). Conversely, overexpression of ISG15 failed to increase the cell fibrosis in TGFβR1 knockout cells (Figure 9A-C). Similar findings were observed in cisplatin stimulated TGFβR1 KO cells transferred with ISG15 (Figure S10). Additionally, compared to TGFβR1 overexpression alone, co-overexpression of ISG15 and TGFβR1 upregulated the transcriptional levels of fibrotic factors in HK-2 cell treated by TGF-β1 (Figure S11). Collectively, these results suggest that TGFβR1 knockout effectively inhibits the fibrosis-promoting effect of ISG15.

## Discussion

ISG15 is the earliest identified ubiquitin-like protein induced by interferons, acting both in a free form and as a protein modifier. ISG15 and the members of the enzymatic cascade that mediate ISG15 conjugation (ISGylation) are strongly induced by type I interferons 17, 20. ISG15 also responds to viral and bacterial infections, lipopolysaccharide, retinoic acid and certain genotoxic stressors in addition to type I interferons 17, 39. Despite its similarities to ubiquitin, ISG15's biological function is still poorly understood; however, ISG15 appears to play important roles in various biological and cellular functions, including innate immunity, anti-viral/bacterial infections, protein turnover, and tumorigenesis 22. Moreover, a recent study suggests a novel DRD4-ISG15-NOX4 axis in the progression of AKI 40. The results showed that dopamine D4 receptor (DRD4) was downregulated in AKI mice, which downregulated ISG15 expression, resulting in decreased ISGylation of NADPH oxidase 4 (NOX4) that competitively inhibited its ubiquitination and caused degradation of NOX4 40.

In this study, we established various animal models of kidney injury and collected samples at multiple time points. The results showed that in the early stages of kidney injury, AKI, knockout of ISG15 ameliorated renal function and pathological change (Figure 2 and Figure S4). Moreover, in the later stages of kidney injury, AKI-to-CKD transition, ISG15 knockout also mitigated renal damage induced by IRI, and UUO (Figure 2-3). Additionally, *in vitro* studies showed that overexpression of ISG15 with modification capability (ISG15GG) promoted cell fibrosis and damage induced by HR, cisplatin or TGFβ1, while overexpression of the free form of ISG15 (ISG15AA) had no effect on cell fibrosis induced by these stimuli (Figure 4-5, Figure S6). These findings collectively underscore the predominant contribution of ISG15 to the initiation and progression of AKI.

IRI, cisplatin, and UUO treatments may induce kidney injury in mice through distinct pathways and with different progression rates. IRI induces kidney damage more rapidly compared to cisplatin- or UUO-induced kidney injury 41-43. Although it is challenging to explain the effects of ISG15 in these kidney injuries using a single mechanism, our data nevertheless indicate that ISG15 modulates renal fibrosis, a common pathway influencing kidney injury in these models. Future efforts to elucidate the distinct and potentially overlapping pathways through which ISG15 operates in kidney injury initiated by different injuries are desired.

To date, hundreds of putative targets of ISGylation have been identified. However, the functional consequence of ISGylation is still poorly understood. Unlike ubiquitylation, ISGylation does not appear to directly target proteins for proteasome-mediated degradation 44. It has been found that ISG15 can compete with ubiquitin for ubiquitin binding sites, thereby indirectly regulating protein degradation 45. In addition, ISG15-ubiquitin mixed chains have been observed and may negatively regulate the turnover of ubiquitylated proteins 17. However, for ISGylated proteins studied, only a small fraction of the total protein is modified by ISG15, making it a challenge to understand how much does ISGylation contribute to the overall function of a protein. In our study, *in silico* results suggested interaction between ISG15 and TGFβR1 (Figure 6F and Table S4), whereas no obvious interaction was predicted between ISG15 and another classic fibrosis receptor TGFβR2 (Table S4). Moreover, co-IP assays confirmed that ISG15 ISGylated TGFβR1, which may prevent TGFβR1 degradation by the ubiquitin-proteasome system (Figure 6).

Renal fibrosis is the principal pathological change and common pathway in the progression of AKI to CKD, ultimately leading to end-stage renal disease and renal failure 46. It is the result of maladaptive repair following renal injury and manifests as a progressive process. TGF-β1 is recognized as the most crucial profibrotic factor and the primary signaling pathway driving renal fibrosis 47-49. TGF-β1 is synthesized and secreted by a wide range of cell types throughout the body. Upon secretion, TGF-β1 activates the TGF-β receptor complex, initiating signaling cascades through both canonical (Smad-dependent) and non-canonical (Smad-independent) pathways 46. The canonical pathway plays central regulatory roles in renal fibrosis 46. In the canonical pathway, upon TGF-β1 activation, TGFβR2 recruits and phosphorylates the serine/threonine kinase domain of TGFβR1 within the cytoplasmic region, leading to the activation of TGFβR1 by binding and phosphorylating the GS domain 47, 48. Subsequently, phosphorylated TGFβR1 activates the fibrotic signaling pathway, leading to the nuclear translocation of Smad2/3 and the regulation of fibrotic-related genes such as α-SMA, fibronectin, and collagen, promoting fibrosis 47, 48. Increasing evidences show that TGFβR1 modification provides a new mechanism for the functional regulation of TGF-β responses. By regulating Smad activation and downstream transcription responses, TGFβR1 sumoylation elaborates the TGF-β responses that drives cancer progression 50. Moreover, ubiquitin-specific protease 2a (USP2a) removes K33-linked ubiquitin chains from TGFβR1 and promotes SMAD2 recruitment 51. Deletion or pharmacologic inhibition of USP2a impairs TGFβ-induced EMT and metastasis 51. TGFβR1 is ubiquitinated by multiple E3s, including SMURF1/2, WWP1 and NEDD4-2, which results in the degradation of TGFβR1 and attenuation of TGF-β signaling 52-54. In our study, we found that ISG15 promoted ISGylation level of TGFβR1 (Figure 6). And, knockout of ISG15 led to decreased TGFβR1 and its downstream p-Smad2 levels, reduces transcription levels of fibrotic genes such as α-SMA, Fn1, and Vimentin, ultimately inhibited renal fibrosis (Figure 2-3 and Figure S4, S8). Additionally, renal overexpression of TGFβR1 abolished the protective effect of ISG15 deficiency during kidney injury (Figures 8-9). Taken together, regulation or inhibition of TGFβR1 modification may be a key to treating AKI and the following AKI-to-CKD transition.

In summary, we reported that increased ISG15 and TGFβR1 expression in the models of IRI-, cisplatin- or UUO-induced kidney injury, and improved kidney function was found in *Isg15* KO mice. We identified a novel modification of the key fibrosis receptor TGFβR1, namely ISGylation, which leads to sustained activation of the TGFβR1 signaling pathway, exacerbating renal fibrosis. Our study also provides a proof-of-concept into AKI/AKI-to-CKD treatment through targeting the modification of TGFβR1 (Figure 9D).

## Supplementary Material

Supplementary figures and tables.

## Abbreviations

AKI: acute kidney injury
IRI: ischemia-reperfusion injury
BIRI: bilateral ischemia-reperfusion injury
UIRI: unilateral ischemia-reperfusion injury
ISG15: Interferon-stimulated gene 15
UUO: unilateral ureteral obstruction
CKD: chronic kidney disease
TGF-β1: transforming growth factor-β1
TGFβR2: type II TGF-β receptor
TGFβR1: type I TGF-β receptor
α-SMA: α-smooth muscle actin
Fn1: fibronectin 1
CREA: creatinine
BUN: blood urea nitrogen
qPCR: quantitative real-time polymerase chain reaction
WT: wildtype
co-IP: co-Immunoprecipitation
ESRD: end-stage renal disease
