# Supplementary material for: ISG15 accelerates acute kidney injury and the subsequent AKI-to-CKD transition by promoting TGFβR1 ISGylation
Source: Theranostics. 2024 Jul 22;14(11):4536–53. doi: 10.7150/thno.95796 (PMC11303071; doi:10.7150/thno.95796)
Supplement: Supplementary file 1 — Supplementary figures and tables. [file thnov14p4536s1.pdf]

Supplementary Materials

for

ISG15 accelerates acute kidney injury and the subsequent AKI-to-CKD transition by promoting TGFβR1 ISGylation

Na Cui[1#], Chengyu Liu[2#], Xiang Tang[1], Liangliang Song[1], Zixuan Xiao[3], Yihao Zhou[4], Chen Wang[1], Yancai Wu[1], Chentai Peng[1], Yuxia Liu[4], Ling Zheng[4], Xinran Liu[1], Kun Huang[1], Hong Chen[1]

[1]Tongji School of Pharmacy, Huazhong University of Science and Technology, Wuhan, China, 430030

[2]Department of Transfusion Medicine, Wuhan Hospital of Traditional Chinese and Western Medicine, Tongji Medical College, Huazhong University of Science and Technology, Wuhan, China, 430000

[3]ISA Wenhua Wuhan High School, Fenglin Road, Junshan New Town, Wuhan Economics & Technological Development Zone, Wuhan, Hubei, China, 430119

[4]Hubei Key Laboratory of Cell Homeostasis, College of Life Sciences, Wuhan University, Wuhan, China, 430072

Corresponding authors

Hong Chen, Ph.D.

Tongji School of Pharmacy

Huazhong University of Science and Technology

Wuhan, China, 430030

hongchen2017@hust.edu.cn

This supplementary file has 11 figures and 4 tables.

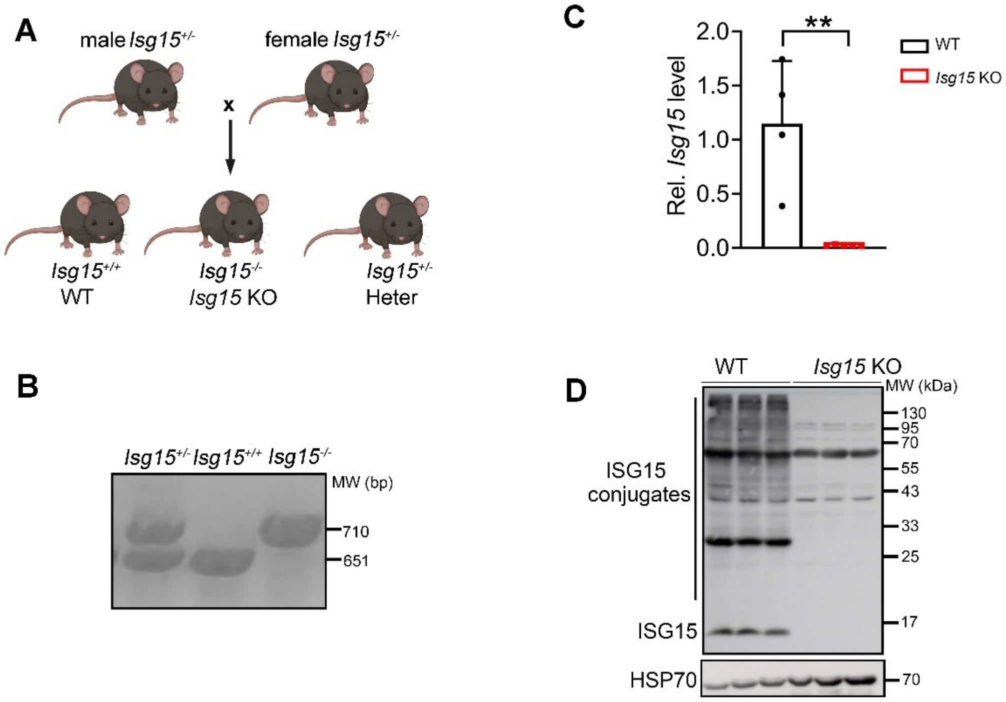

Figure S1. *Isg15* KO mice were successfully generated. (A) Experimental design for breeding. **(B)** Genotyping of WT and *Isg15* KO mice. **(C)** qPCR result of *Isg15* mRNA level in the kidneys of WT (n = 4) and *Isg15* KO (n = 5) mice. **(D)** Western blots of ISG15, HSP70 in the kidney of WT and *Isg15* KO mice. $^{**}P < 0.001$.

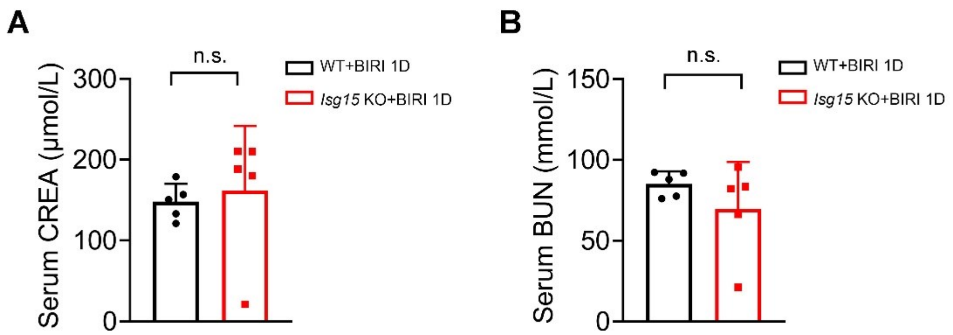

Figure S2. *Isg15* KO showed no effect on mice at 1 day after bilateral IRI. (A-B) Serum creatinine (A) and BUN (B) levels in WT (n = 5) and *Isg15* KO mice (n = 5) at bilateral ischemia reperfusion injury day 1(BIRI 1D). n.s., not significant.

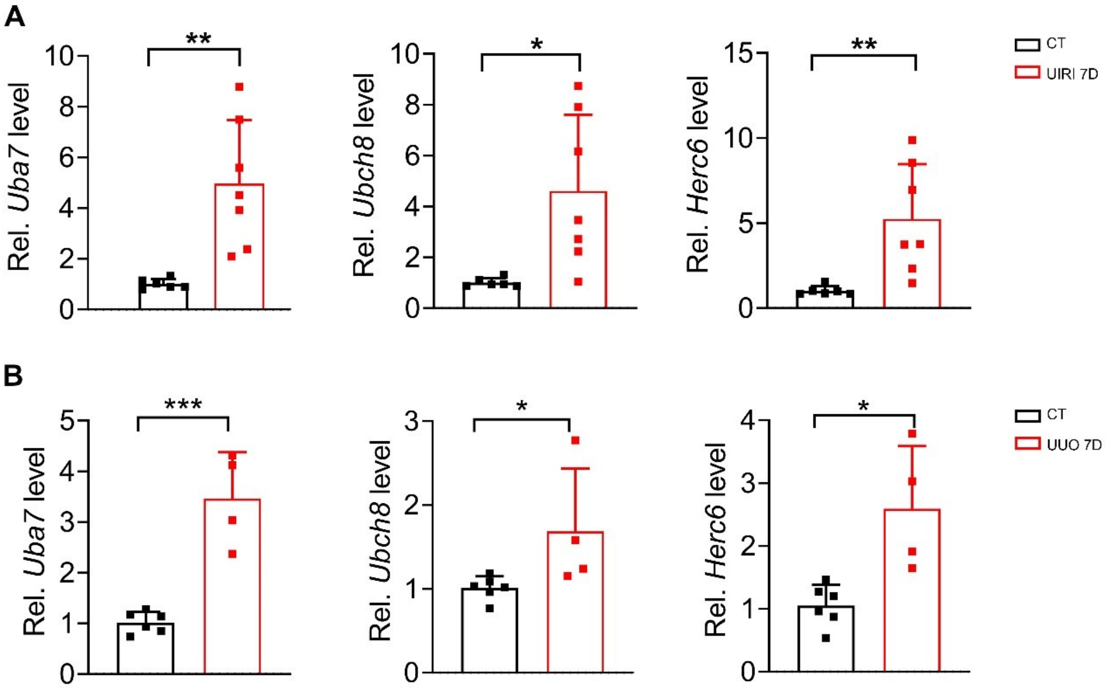

Figure S3. *Uba7*, *Ubch8*, *Herc6* mRNA levels increases in the kidney of mice post UIRI, or UUO 7D. (A) qPCR result of *Uba7*, *Ubch8*, *Herc6* in the kidneys of control (n = 6) and UIRI-injured (n = 7) mice. **(B)** qPCR result of *Uba7*, *Ubch8*, *Herc6* in the kidneys of control (n = 6) and UUO-injured (n = 4) mice. *$P < 0.05$; **$P < 0.01$; ***$P < 0.001$.

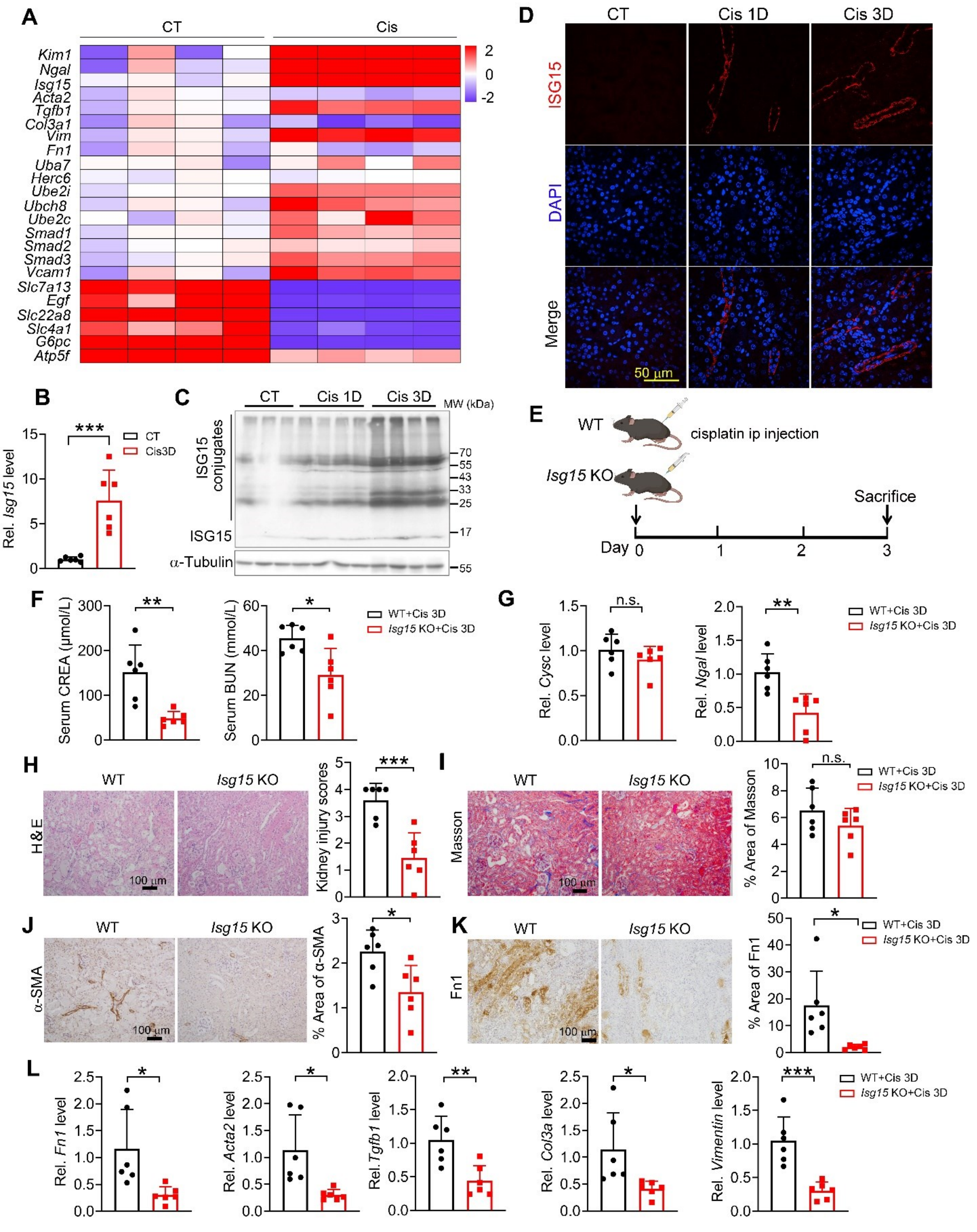

Figure S4. ***Isg15*** **knockout inhibits cisplatin induced kidney injury. (A)** RNA-seq data of

ISG15 and its related ligase genes, and fibrotic genes in the kidneys of cisplatin injured mice. **(B-C)** qPCR (B) and WB (C) results of *Isg15* of non-injured (CT) or cisplatin injured mice at indicated times. **(D)** Representative images of ISG15 (red), and DAPI (blue) staining of the kidney of CT or cisplatin injured mice at indicated times. **(E)** Experimental design chart of cisplatin injury. **(F)** Serum creatinine (left) and BUN (right) levels for indicated experimental groups at 3 days after cisplatin injury. **(G)** qPCR results of *Cysc*, *Ngal* in the kidneys of WT and *Isg15* KO mice after cisplatin injury. **(H)** Representative H&E images (left) with injury scores (right) in the kidney of WT and *Isg15* KO mice after cisplatin injury. **(I-K)** Representative images and quantitative results of Masson staining (I), immunostaining for α-SMA (J), Fn1 (K) of the kidney of WT and *Isg15* KO mice after cisplatin injury. Scale bar = 100 μm. **(L)** qPCR results of *Fn1*, *Acta2*, *Tgfb1*, *Col3a* and *Vimentin* in the kidney of WT and *Isg15* KO mice after cisplatin injury. WT+Cis 3D, n = 6; *Isg15* KO+Cis 3D, n = 6. *$P < 0.05$; **$P < 0.01$; ***$P < 0.001$; n.s., not significant.

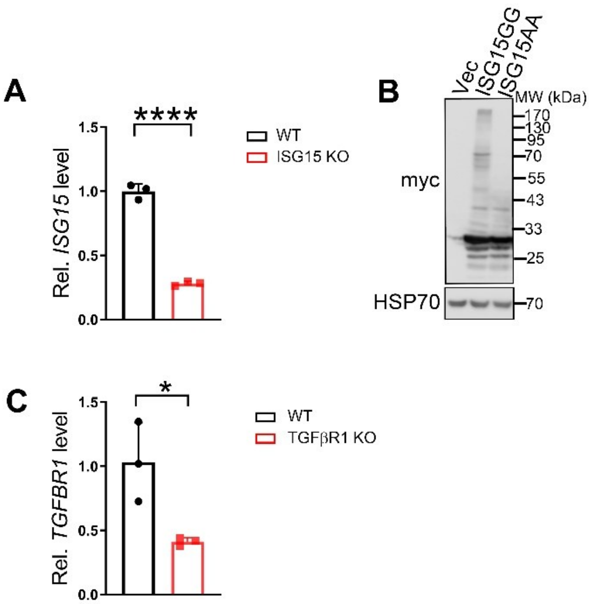

Figure S5. Knockout of ISG15 and TGFβR1, and overexpression of ISG15 in cultured HK-2 cells. (A) qPCR result of *Isg15* in the HK-2 cells. **(B)** Western blots of myc, HSP70 in HK-2 cells when overexpressed myc-1SG15GG, myc-1SG15AA. **(C)** qPCR result of *TGFBR1* in the HK-2 cells. *$P < 0.05$; ****$P < 0.0001$.

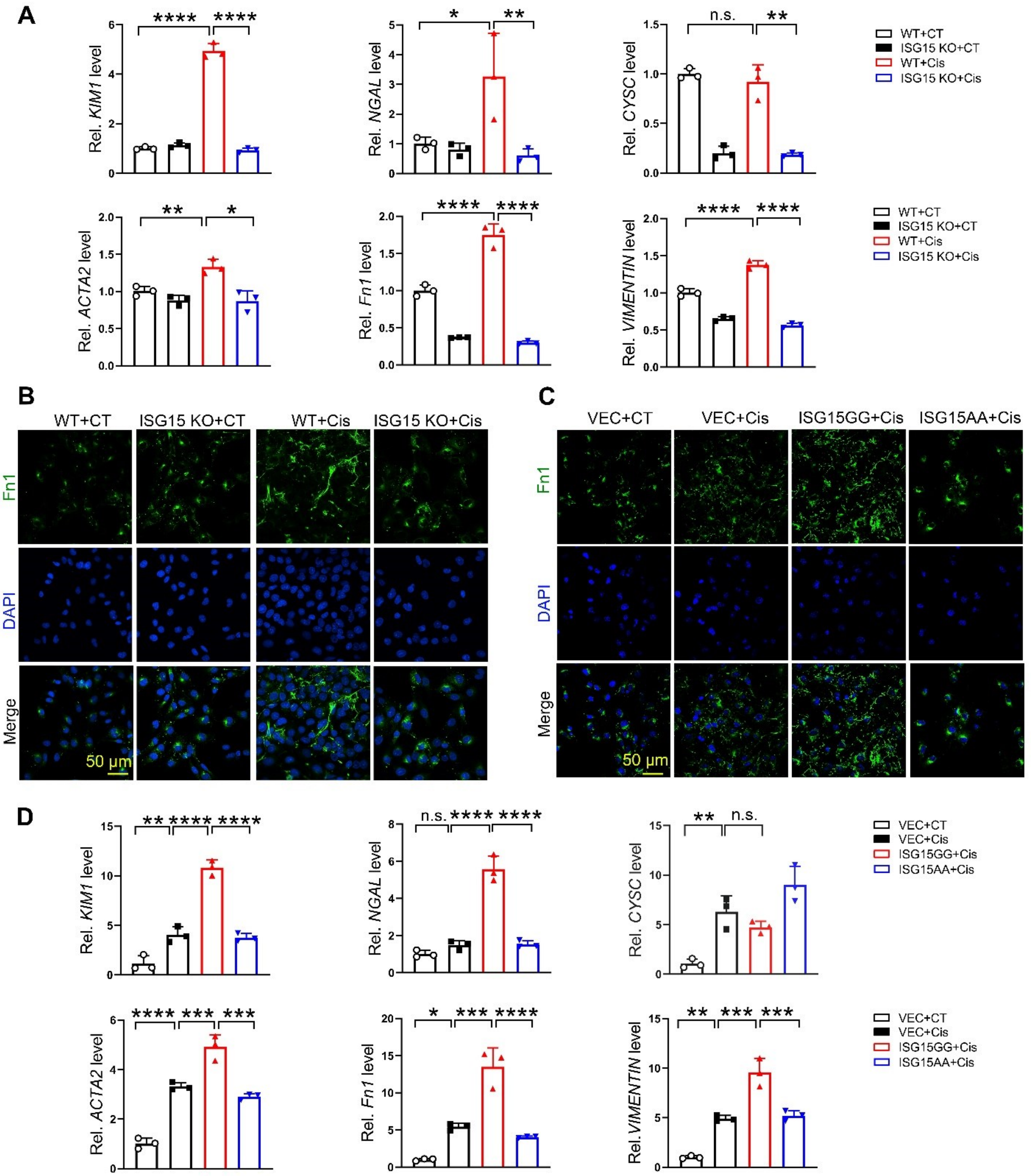

Figure S6. ISG15 accelerates cisplatin induced fibrosis in cultured HK-2 cells. (A) qPCR results of *KIM1*, *NGAL, CYSC*, *ACTA2*, *Fn1* and *VIMENTIN* in ISG15 knockout HK-2 cells with or without cisplatin treatment. **(B)** Representative Fn1 images in ISG15 knockout HK-2 cells with

or without cisplatin treatment. Scale bar = 50 µm. **(C)** Representative Fn1 images in ISG15 overexpressed HK-2 cells with cisplatin treatment. Scale bar = 50 µm. **(D)** qPCR results of *KIM1*, *NGAL, CYSC*, *ACTA2*, *Fn1* and *VIMENTIN* in ISG15 overexpressed HK-2 cells with cisplatin treatment. The experiments were repeated three times, and at least three biological replicates per group were used. *$P < 0.05$; **$P < 0.01$; ***$P < 0.001$; ****$P < 0.0001$; n.s., not significant.

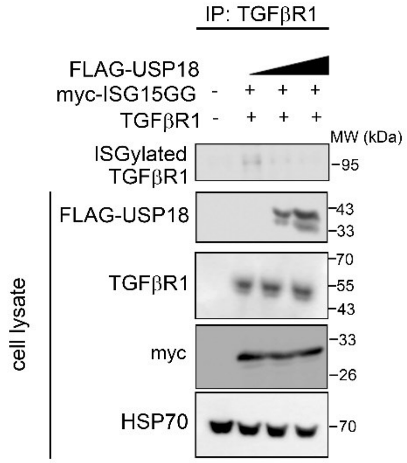

Figure S7. Overexpression of USP18 suppressed TGFβR1 ISGylation. Western blots of TGFβR1, myc, FLAG and HSP70 in the HEK293T cells.

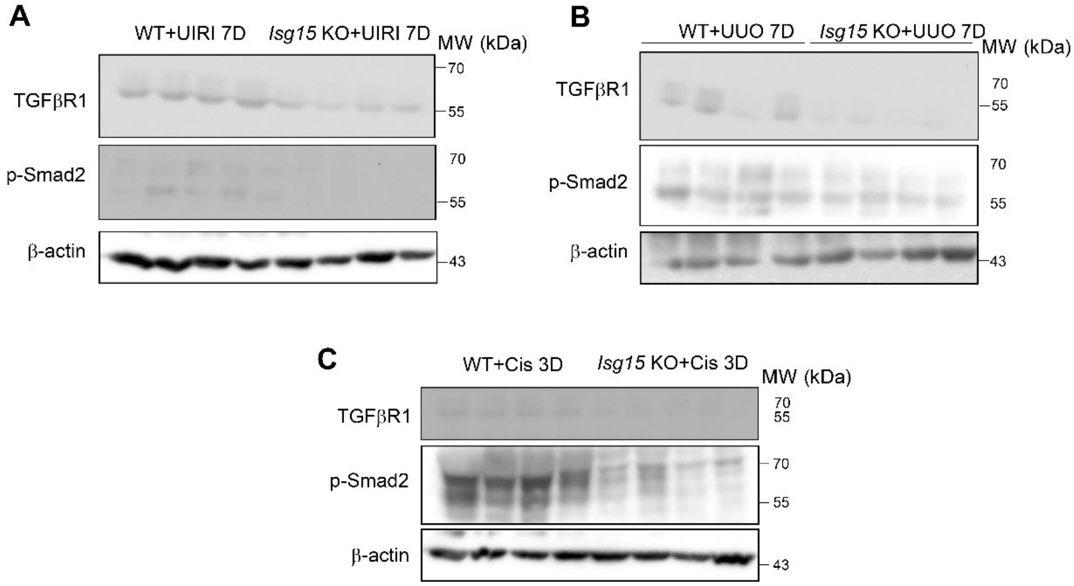

Figure S8. *Isg15* KO decreased TGFβR1 and p-Smad2 levels in the kidneys of UIRI, UUO, cisplatin treated mice. (A-C) Western blots of TGFβR1, p-Smad2, β-actin in the kidney of WT (n = 4) and *Isg15* KO (n = 4) mice post UIRI (A), UUO (B), cisplatin (C) injury.

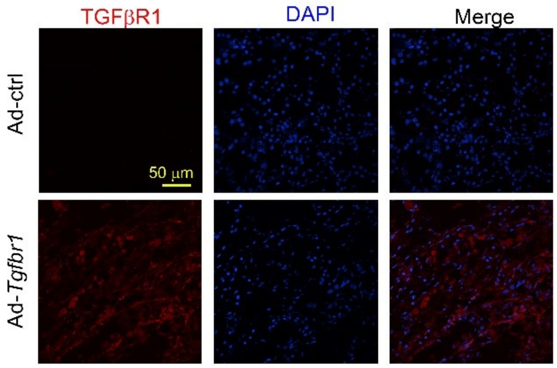

Figure S9. Overexpression of TGFβR1 in the kidney of mice by adenovirus injection. Representative TGFβR1 images in the kidney of TGFβR1 overexpressed mice by adenovirus injection. Scale bar = 50 μm.

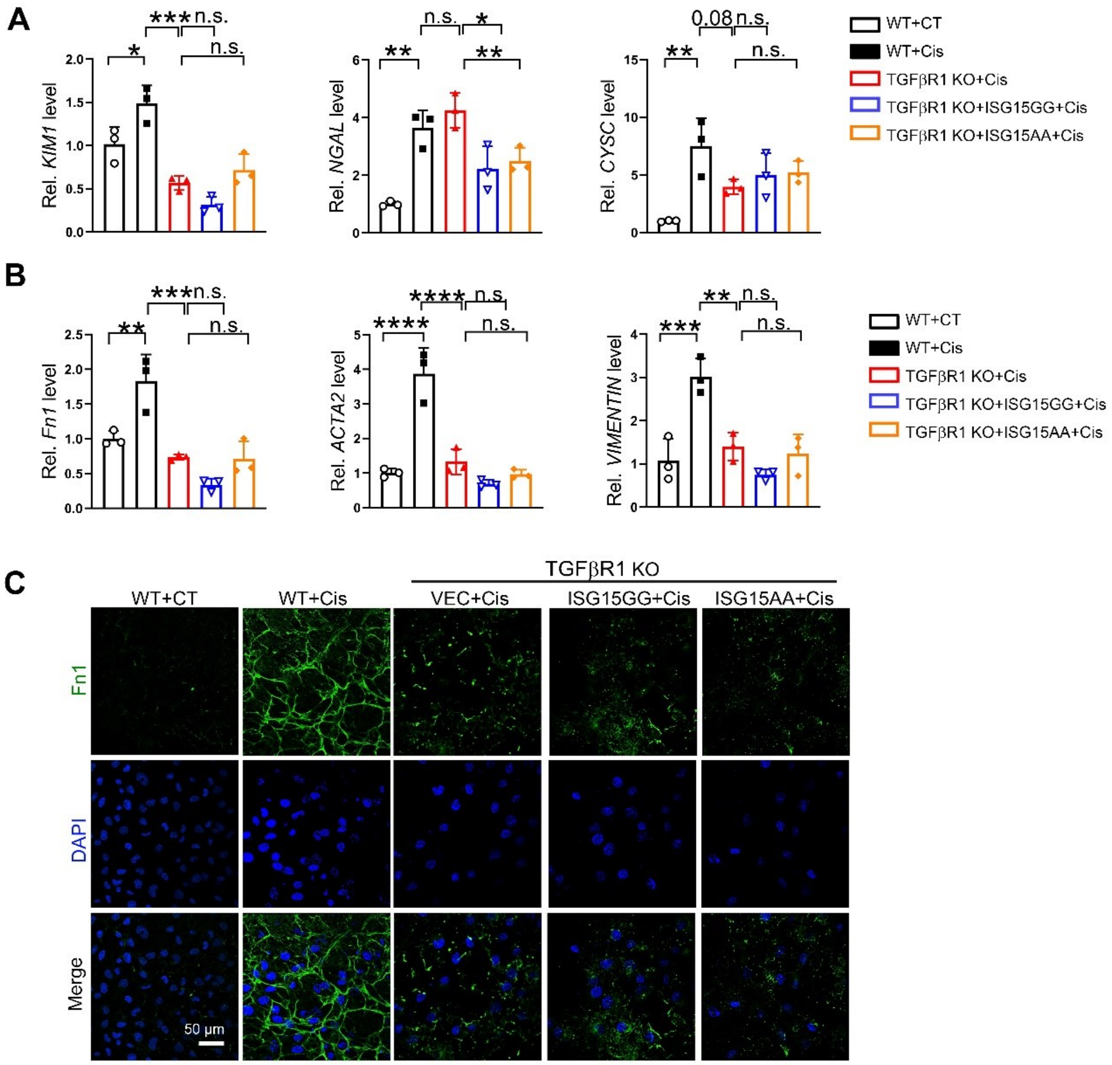

Figure S10. ISG15 aggravates renal cell damage *via* TGFβR1. (A-B) qPCR results of *KIM1*, *NGAL, CYSC*, *ACTA2*, *Fn1* and *VIMENTIN* in TGFβR1 knockout HK-2 cells overexpression of ISG15 under ciplatin treatment. **(C)** Representative Fn1 images in TGFβR1 knockout HK-2 cells overexpression of ISG15 under ciplatin treatment. Scale bar = 50 μm. The experiments were repeated three times, and at least three biological replicates per group were used. **P* < 0.05; ***P* < 0.01; ****P* < 0.001; *****P* < 0.0001; n.s., not significant.

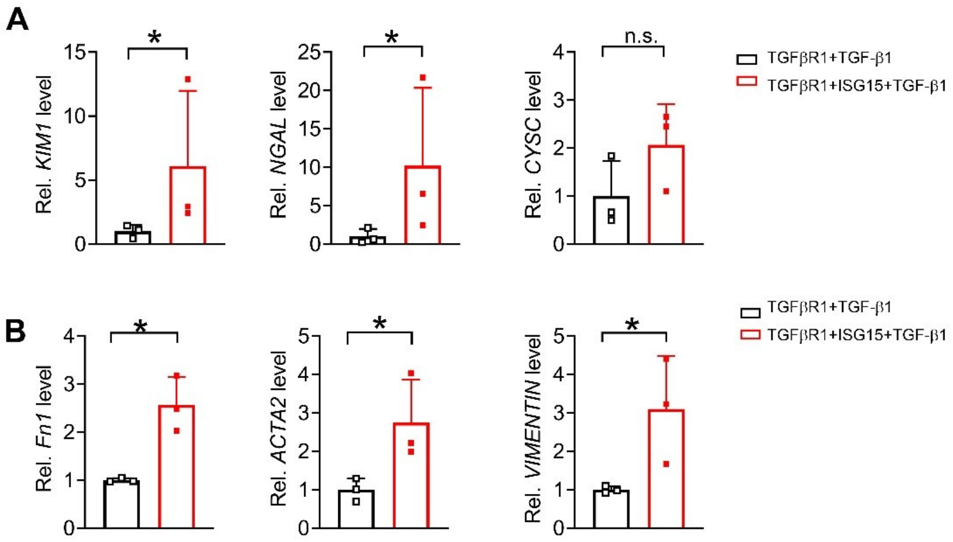

Figure S11. ISGylation of TGFβR1 enhances its fibrotic activity. (A-B) qPCR results of *KIM1*, *NGAL*, *CYSC*, *ACTA2*, *Fn1* and *VIMENTIN* in HK-2 cells transfected with TGFβR1 alone, or both TGFβR1 and ISG15 under TGF-β1 treatment. The experiments were repeated three times, and at least three biological replicates per group were used. *$P < 0.05$; n.s., not significant.

Table S1. Primers for plasmids constructed in the study.

Primer name	Sequence (5'-3')
FLAG-TGFβR1	Forward: ATTGTCGACCATGGAGGCGGCGGTCGCTGCT
	Reverse: ATTGCGGCCGCTTACATTTTGATGCCTTCCT
HA-E1 (UBA7)	Forward: AGGACTTGAATTTAGAGATCTATATGGATGCCCTGGACGCTTC
	Reverse: GCTGGGTCTAGATAT CTCGAGTCACAGCTCATAGTGCAGAG
HA-E2 (UBCH8)	Forward: AGGACTTGAATTTAG AGATCTATATGATGGCGAGCATGCGAGT
	Reverse: GCTGGGTCTAGATAT CTCGAGTTAGGAGGGCCGGTCCACTC
HA-E3 (HERC5)	Forward: AGGACTTGAATTTAGAGATCTATATGGAGCGGAGGTCGCGGAG
	Reverse: GCTGGGTCTAGATAT CTCGAGTCAGCCAAATCCTCTGTTGT
**Primers used for genotyping**	
Isg15 primer1	Forward: TGGACTACATAGCAAAGACTACTTC
	Reverse: GCCCGAGTAAGCTGATAGAGG
Isg15 primer2	Forward: CTCTTGTTTGCTTGTCCCTCTCC
	Reverse: GCCCGAGTAAGCTGATAGAGG

Table S2. Antibodies used in this study.

**Antibody**	**Catalog number**	**Company**
α-Tubulin (WB)	AF0001	Beyotime
ISG15 (WB, IF)	PA5-79523	Invitrogen
Fn1(IHC, IF)	Sc-18825	Santa Cruz
a-SMA (IHC)	14395-1-AP	Proteintech
myc (WB)	AE009	Abclonal
FLAG (WB)	F1804	Sigma
β-actin (WB)	A5316	Sigma
TGFβR1 (WB)	Ab235578	Abcam
UBA7 (WB)	146504	Absin
HERC5 (WB)	22692-1-AP	Proteintech

WB, Western blotting; IHC, Immuno-histochemistry; IF, Immunofluorescence.

Table S3. qPCR primers used in this study

Gene	Forward	Reverse
M *Isg15*	GGGGGAGTATGGCCTAAAGC	CCAACACTGGCTCTGGATGG
M *Tgfbr1*	AAAAGCAGTCAGCTGGCCTT	ATGACAGTGCGGTTATGGCA
M *Kim1*	ACATATCGTGGAATCACAACGAC	ACTGCTCTTCTGATAGGTGACA
M *Ngal*	TGGCCCTGAGTGTCATGTG	CTCTTGTAGCTCATAGATGGTGC
M *Vimentin*	GGCTGCGAGAGAAATTGCAG	CGTTCAAGGTCAAGACGTGC
M *Col3a*	CTGTAACATGGAAACTGGGGAAA	CCATAGCTGAACTGAAAACCACC
M *Tgfb1*	TGGCCAGATCCTGTCCAAAC	GTTGTACAAAGCGAGCACCG
M *Fn1*	GCCTGAACCAGCCTACAGAT	AGCTTAAAGCCAGCGTCAGA
H *ISG15*	ACAGCCATGGGCTGGGA	CCTTCAGCTCTGACACCGAC
H *TGFBR1*	GGTTCCGTGAGGCAGAGATT	CTGAGTCCAAGTACCATTGTCTTTA
H *KIM1*	TGGCAGATTCTGTAGCTGGTT	AGAGAACATGAGCCTCTATTCCA
H NGAL	GACAACCAATTCCAGGGGAAG	GCATACATCTTTTGCGGGTCT
H *CYSC*	GTCGGCGAGTACAACAAAGC	CACCCCAGCTACGATCTGC
H *Fn1*	ACAAGCATGTCTCTCTGCCA	CCAGGGTGATGCTTGGAGAA
H *VIMENTIN*	GCGAGGAGAGCAGGATTTCT	ACCAGAGGGAGTGAATCCAGA
H *ACTA2*	AGCCATTGAAAAGGCAGGGA	GGAGCTTGTCCTTCACCTCC
M *Rn18s*	CTCAACACGGGAAACCTCAC	CGCTCCACCAACTAAGAACG

Table S4. Protein-protein interaction (PPI) modeling of human TGFβR1, TGFβR2, and ISG15

PPI	Interface area (Å²)	$\Delta^{i}G$ (kcal/mol)
ISG15-TGFβR1	1816.3	-7.3
ISG15-TGFβR2	1202.4	-1.1

Interface area, accessible surface area; $\Delta^{i}G$ Solvation energy effect, kcal/mol.
